# Impact of substrate curvature on grazing-incidence small-angle X-ray scattering signal: theory and example of Ag thin-film growth

**DOI:** 10.1107/S1600576725010726

**Published:** 2026-02-01

**Authors:** Michał Kamiński, Bärbel Krause, Gregory Abadias, Alessandro Coati, Yves Garreau, Anny Michel, Andrea Resta, Karan Solanki, Alina Vlad, David Babonneau

**Affiliations:** a Institute of Photon Science and Synchrotron Radiation (IPS), Karlsruhe Institute of Technology (KIT), Karlsruhe, Germany; b Université de Poitiers, ISAE-ENSMA, CNRS, PPRIME, Poitiers, France; c Synchrotron SOLEIL, Saint Aubin, France; d Laboratoire Matériaux et Phénomenes Quantiques, Université Paris Cité, Paris, France; Montanuniversität Leoben, Austria

**Keywords:** grazing-incidence small-angle X-ray scattering, GISAXS, substrate curvature, thin-film growth, stress, real-time techniques

## Abstract

Grazing-incidence small-angle X-ray scattering (GISAXS) is a technique of choice for providing information about the morphology of nano- and micro-structures at surfaces and interfaces, also in real time. The geometry of the sample, in particular its curvature, has an impact on the observed X-ray scattering signal. There are a multitude of systems with sophisticated geometries (including curvature), ranging from electronic devices on flexible substrates to biological membranes, for which GISAXS could provide valuable information. Therefore, in this work the effect of the sample geometry on the GISAXS signal is addressed. More specifically the influence of the substrate curvature and extent along the X-ray beam is considered. The analytical expressions accounting for the effects of those two geometrical parameters are provided, and the way to include them in the analysis of GISAXS patterns is described. The calculations reveal that no corrections are needed for small samples (length over distance to the detector ratio smaller than 1%) and radius of curvature |*R*| > 50 m. These results allow for a combination of GISAXS with substrate curvature measurements. The latter technique is a non-destructive *in situ* and real-time method providing information about the intrinsic stress in a thin film during its growth. Morphological information from GISAXS is supposed to complement this stress information. Herein this methodology is applied to the growth of Ag thin films deposited by magnetron sputtering with N_2_ plasma additive. The analysis of the GISAXS pattern obtained from the sample, which bends during the deposition, provided morphological parameters of the growing film. This methodology can be useful for understanding of the mechanisms at the nanoscale leading to the observed stress state. The ability to perform GISAXS on curved substrates enables its application to more complex systems.

## Abstract 2

The paper formally addresses the influence of the substrate curvature and size on the grazing-incidence small-angle X-ray scattering (GISAXS) signal. Using the established theory, GISAXS can be combined with substrate-curvature-based stress measurements, which is demonstrated on the example of Ag thin-film growth.

## 1. Introduction

Grazing-incidence X-ray scattering (GIXS) techniques are powerful tools for investigation of surfaces and interfaces at the nanoscale (Renaud *et al.*, 2009 ▸; Hexemer & Müller-Buschbaum, 2015 ▸; Birkholz, 2006 ▸; Tolan, 1999 ▸; Lazzari, 2002 ▸; Pietsch *et al.*, 2004 ▸). The use of grazing-incidence angles limits the penetration depth of X-rays, so the measured signal comes only from several nanometres of the topmost layer. Two widely used methods are grazing-incidence small-angle X-ray scattering (GISAXS) and X-ray reflectivity (XRR). GISAXS yields information about the morphology of the surface, including the size, shape and distribution of nano-objects (Levine *et al.*, 1989 ▸; Renaud *et al.*, 2009 ▸). The XRR signal is related to the out-of-plane scattering length density profile of the sample and thus yields information about the thickness, roughness and electron density profile of the sample near the interface (Daillant & Gibaud, 1999 ▸). GIXS techniques are often used for complex systems where a surface or an interface plays a central role. These methods yield a comprehensive understanding of the investigated system (Müller-Buschbaum, 2014 ▸; Jaksch *et al.*, 2019 ▸; Wyon, 2010 ▸) and can be used also for real-time (Krause *et al.*, 2023 ▸; Jankowski *et al.*, 2021 ▸; Held *et al.*, 2024 ▸; Held *et al.*, 2025 ▸) and *operando* (Yang *et al.*, 2020 ▸) studies.

GISAXS, often in sophisticated experimental configurations, is used to address manifold research questions. Among others, this includes studies of liquid–liquid interfaces (Takiue & Aratono, 2024 ▸; Huerre *et al.*, 2018 ▸; Fink *et al.*, 2023 ▸; Hemmerle *et al.*, 2024 ▸), thin-film growth (Shao *et al.*, 2023 ▸; Wieser *et al.*, 2023 ▸; Betker *et al.*, 2023 ▸; Held *et al.*, 2025 ▸), and self-assembly of nanostructures (Gibaud *et al.*, 2003 ▸; Ree, 2014 ▸; Weidman *et al.*, 2016 ▸; Kang *et al.*, 2023 ▸), catalyst materials (Chang *et al.*, 2024 ▸), proteins (Hofmaier *et al.*, 2023 ▸) and micrometre-sized objects (Maiti *et al.*, 2024 ▸). Given the increasing complexity of the studied systems, in this work we will systematically address a possible influence of the geometry of the sample (its size and curvature) on the measured scattering signal.

Specifically, we aim at combining GISAXS with substrate curvature measurements, with both techniques performed simultaneously in real time during the growth of Ag thin films by sputter deposition. This approach yields an in-depth understanding of the processes during and after the nano­structure formation, which cannot be provided by *ex situ* diagnostics because of post-growth morphological changes such as dewetting (Zapata *et al.*, 2024 ▸; Sarakinos *et al.*, 2024 ▸; Rumsby *et al.*, 2024 ▸; Cueva & Carretero, 2025 ▸; Thompson, 2012 ▸; Abduvalov *et al.*, 2023 ▸; Jacquet *et al.*, 2018 ▸) due to high Ag atom mobility. Microstructural changes occurring in the course of film growth are associated with the development of intrinsic stress, which causes bending of the film/substrate system to maintain mechanical equilibrium. If the substrate is thin enough, then the change in curvature can be accurately measured (Stoney, 1909 ▸; Freund *et al.*, 1999 ▸). This non-destructive and experimentally simple method provides information about the stress state during the growth and in the as-deposited structure (Abadias *et al.*, 2018 ▸). Moreover, the features exhibited by the stress curve carry information about the structural evolution of the film. For instance, metal layers growing on a weakly interacting substrate (such as oxides or van der Waals materials) follow a Volmer–Weber mode, resulting in discontinuous layers (3D islands) in the early growth stages. It has been shown that the onset of film continuity, *i.e.* the end of the coalescence stage between islands, coincides with a tensile peak maximum (Abadias *et al.*, 2015 ▸). This makes substrate curvature measurements an ideal method for quick assessment of the influence of particular deposition parameters on the thin-film growth, with possible industrial relevance. With our methodology we aim to establish a correlation between the observed stress signal and underlying film formation mechanisms. The information about evolution of film morphology is provided by GISAXS. Since a combination of GISAXS and substrate curvature measurements has not been reported so far, we present here a systematic evaluation of the impact of substrate curvature on the scattering signal and prove the feasibility and usefulness of this experimental approach.

The importance of geometrical effects as a possible source of increased experimental uncertainties was mentioned in the paper by Yang (2005 ▸) and further discussed in more detail by Smilgies (2009 ▸) and Wernecke *et al.* (2014 ▸). The latter article describes the possibility of using GISAXS as a nanometrology tool for determination of the pitch of polymer gratings. A precise determination of the sample-to-detector distance (*d*) was identified as a major prerequisite for accurate size measurement of the nano-objects. Smilgies discusses the smearing due to the finite size of the footprint of the incident beam on the sample. Using analytical formulas and GISAXS simulations, we will show that the sample length along the beam can lead to distortions of the GISAXS signal with respect to that calculated for a point-like sample.

The impact of sample curvature on the XRR signal was studied by several groups (Festersen *et al.*, 2018 ▸; Konovalov *et al.*, 2022 ▸; Gao *et al.*, 2022 ▸; Belova *et al.*, 2023 ▸). In particular, Festersen *et al.* have studied systematically its impact on XRR performed on the curved surface of droplets (Festersen *et al.*, 2018 ▸). Further experimental and theoretical studies allowing for an interpretation of the XRR data from the curved surfaces of liquid metal catalysts were performed by Konovalov *et al.* (2022 ▸). Belova *et al.* (2023 ▸) have shown that, because of the curvature, it is possible to extract not only the out-of-plane scattering length density profile but also information about the in-plane structure of thin films using the XRR signal.

Many systems where a curvature of the sample may possibly occur have been studied experimentally with GISAXS. First of all, the use of thin or elastically soft wafers naturally introduces a possibility of unintended curvature of the sample during measurements. Such substrates are routinely used for fabrication of electronic circuits or devices (Kang *et al.*, 2005 ▸; Shimoto *et al.*, 2004 ▸; Vella *et al.*, 2009 ▸; Buencuerpo *et al.*, 2012 ▸; Yordanov & Angelopoulos, 2013 ▸; Rani *et al.*, 2023 ▸; Mönch *et al.*, 2011 ▸). Curved substrates may also be used deliberately (Sun *et al.*, 2005 ▸). Increasing interest in flexible electronics is resulting in the use of curved or flexible substrates (Lucarini *et al.*, 2021 ▸; Wei *et al.*, 2020 ▸). The properties of biological membranes are inherently related to their curvature (Lipowsky, 1991 ▸; McMahon & Boucrot, 2015 ▸). Among other methods, they are also investigated using GISAXS (Paracini *et al.*, 2023 ▸; Montis *et al.*, 2020 ▸), and their geometry can potentially have an impact on the observed scattering signal. The surface curvature has also been observed to influence ordering of molecules (Hsieh *et al.*, 2012 ▸; Beltrán-Heredia *et al.*, 2019 ▸). In addition, curvature of the substrate occurs in investigation of nucleation and growth on liquid droplets (Sartori *et al.*, 2022 ▸; Konovalov *et al.*, 2022 ▸; Belova *et al.*, 2023 ▸) and liquid-crystal films suspended on the grids used for biosensing (Popov *et al.*, 2017 ▸).

In this article, we aim to identify the conditions under which geometrical effects have a significant impact and must be considered in the analysis of the scattering signal. If that is the case, those effects can be included in the calculation of the theoretical scattering pattern. We consider both convex (compressive stress) and concave (tensile stress) samples, as these cases are observed during the Volmer–Weber growth of polycrystalline metal films depending on the stage of the deposition (Koch, 1994 ▸).

The paper is structured as follows. In Section 2, a theoretical framework is presented, which allows us to account for geometrical effects related to the sample length and its curvature. This part is followed by the results of numerical calculations of GISAXS patterns for flat and curved samples (Section 3). Finally, a set of experimental data from real-time experiments combining GISAXS and curvature measurements during sputter deposition of Ag on a thin Si substrate is presented (Section 4). The results of the GISAXS analysis are shown and demonstrate the feasibility of its combination with substrate curvature monitoring to allow for understanding the morphological origins of the intrinsic stress in thin films.

## 2. Theory

### 2.1. Description of the system

The scattering signal is a function of the incident (α_i_) and exit scattering angles (α_f_ and 2Θ_f_). Thus, in the following sections the impact of sample curvature on incident and exit angles is discussed. Here the focus is on the GISAXS technique, but the formalism can be also used for XRR (for which 2Θ_f_ = 0 and α_f_ = α_i_ in the case of flat samples).

**Figure 1:**
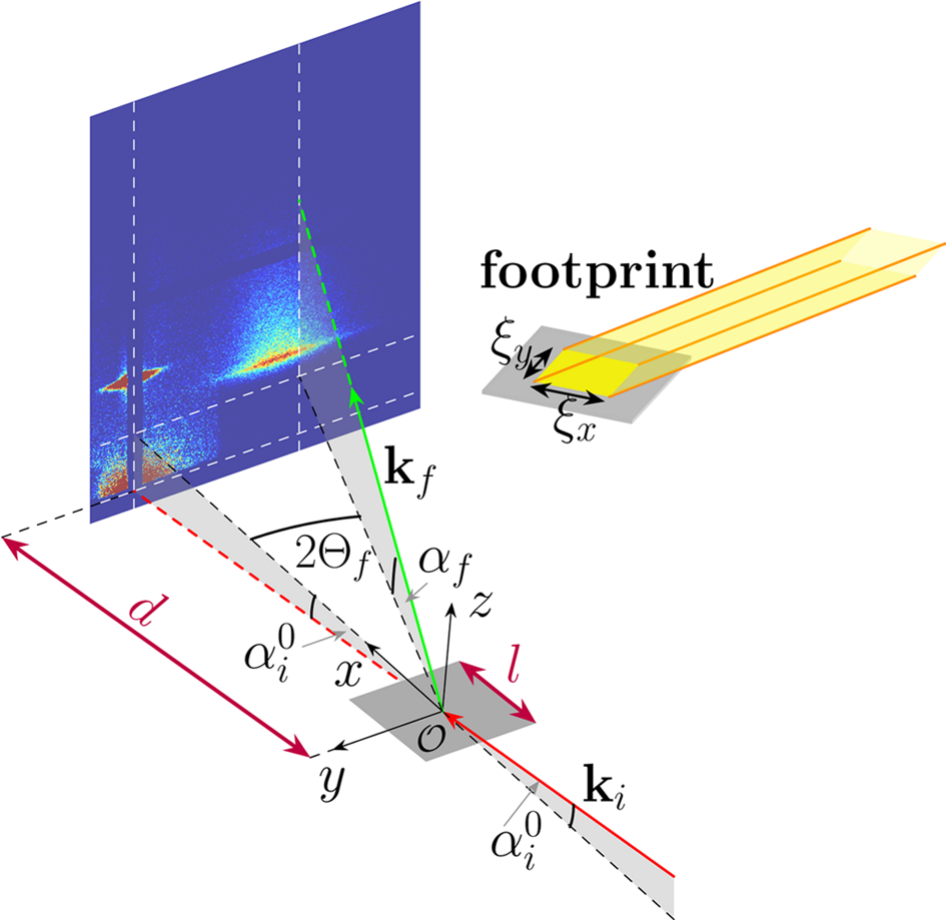
The geometry of the GISAXS experiment for the case of a flat sample. On the right the footprint of the rectangular incident X-ray beam on the sample surface is depicted schematically. For more details see the text.

The geometry of the experiment and the used coordinate system are depicted in Fig. 1 ▸ for GISAXS and the case of flat samples.

The sample coordinate system is defined with respect to the flat sample surface such that the *z* axis is perpendicular to the surface, the *x* axis is along the incident X-ray beam and the *y* axis is perpendicular to the beam, so that it forms a right-handed coordinate system. The centre of the beam coincides with the centre of the sample and the origin of the coordinate system. The footprint of the beam on the sample has a size of ξ_*y*_ × ξ_*x*_ (perpendicular × along the propagation direction of the X-ray beam). The incident angle α_i_ (which is constant and equal to 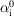 for flat samples) is the angle between the incident wavevector **k**_i_ and the tangent to the sample surface. The vertical exit angle α_f_ is the angle between the exit wavevector **k**_f_ and the tangent to the sample surface. The horizontal exit angle 2Θ_f_ is the angle between the in-plane component of **k**_f_ and the *x* axis. The scattering is elastic, so |**k**_i_| = |**k**_f_| = *k* = 2π/λ, where λ is the wavelength of the X-rays.

In the GISAXS technique the intensity scattered by a nano-object located on a substrate is given by
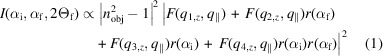
within the distorted wave Born approximation (DWBA) (Renaud *et al.*, 2009 ▸), where 
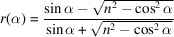
is a Fresnel coefficient, *F* denotes a form factor, *n*_obj_ is the refractive index of the nano-object and *n* is that of the substrate. The components of the scattering vector **q** = **k**_f_ − **k**_i_ are given by (Lazzari, 2002 ▸; Renaud *et al.*, 2009 ▸)
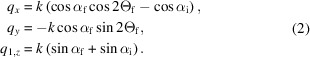
The remaining momentum transfer components used in equation (1) are
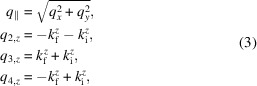
with the *z* components of the wavevectors equal to 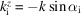 and 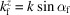.

The scattering pattern recorded at the detector is a superposition of the signal coming from the entire sample surface illuminated by the beam. The curvature of the sample leads to the local variation of the incidence and exit angles across the sample surface. Also, in the case of a flat sample with non-negligible spatial extent the exit angles vary because of the different position of each point of the surface with respect to the detector. The relative change of the angles can be significant, since the absolute values of the incident and exit angles are small in grazing-incidence techniques. The scattering vector **q** = **q**(α_i_, α_f_, 2Θ_f_) is a function of the incident and exit angles. Therefore, the sample geometry leads to the local change of the scattering signal.

**Figure 2:**
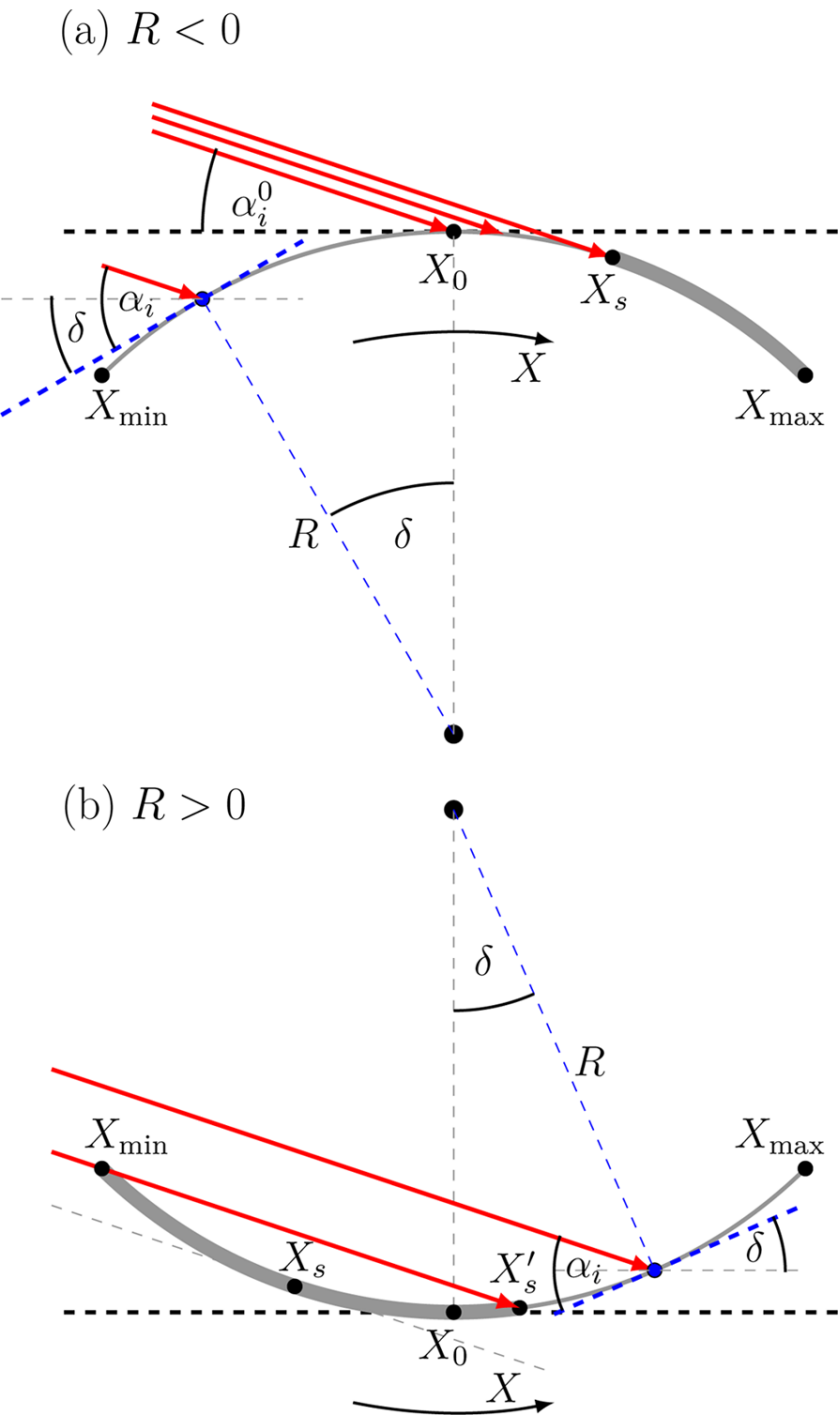
The local incident angle on the curved sample for a (*a*) convex (*R* < 0) and (*b*) concave (*R* > 0) sample. Red arrows indicate selected rays of the incoming beam. It was assumed that the footprint of the beam in its propagation direction is larger than the sample length (ξ_*x*_ > *L*). The dashed black line symbolizes the flat sample. The positions on the surface of the curved sample are parametrized with a curvilinear coordinate *X* with the origin marked by *X*_0_. The local incident angle α_i_ is defined with respect to the tangent at a given point *X* (blue colour). δ is the angle between the radii drawn to the origin (*X*_0_) and to the considered point *X*. For sufficiently high curvatures and small nominal incident angles there is a shadow, marked by thick grey lines in the schemas (for details see text).

As mentioned, the internal stress in the growing thin film can be determined indirectly by monitoring the substrate curvature, which is assumed to be spherical (Leusink *et al.*, 1992 ▸). Therefore we adopt such a form of curvature and limit our consideration to the case of curvature in the direction parallel to the beam, *i.e.* along the **x** direction. Following the work of Briscoe *et al.* (2012 ▸) and Konovalov *et al.* (2022 ▸), this is justified for ξ_*y*_/(2|*R*|) < 10^−3^. The sub-millimetre size of the beam perpendicular to its propagation direction limits the size of the illuminated sample surface in this direction. For example, in our experiment ξ_*y*_ = 150 µm, yielding from the above-mentioned condition |*R*| > 0.075 m. Realistic values due to the stress emerging from nanostructures grown by physical vapour deposition are well above this limit. Thus, since one dimension is effectively neglected, the sample is a part of the side surface of a cylinder with radius *R* and length *l* (Fig. 2 ▸). By convention, negative (*R* < 0) and positive (*R* > 0) radii of curvature correspond to convex and concave shapes, respectively. The incident angle at the apex is denoted by 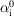 (and equals the incident angle for a flat sample). In order to describe the sample surface we introduce the curvilinear coordinate *X*. The origin of this coordinate corresponds to the centre of the sample. *X* is in the range [−*l*/2; *l*/2] with *l* = *L* if 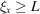 and *l* = ξ_*x*_ if ξ_*x*_ < *L*, where *L* is the geometrical length of the substrate. These coordinates are used to define the local incident and exit angles on the curved sample surface.

### 2.2. Incident angle

In the following two sections we quantify the impact of the sample geometry (curvature 1/*R* and length *l*) on the local incident (Section 2.2) and exit angles (Section 2.3). The curvature of the substrate leads to the local deviation of the incident angle from its value for the flat sample (Fig. 2 ▸). We assume that the sample is fully illuminated by the incident X-ray beam (ξ_*x*_ > *L*). The lowest and highest values of the curvilinear coordinate are then *X*_min_ = −*l*/2 and *X*_max_ = *l*/2, respectively. As one can see in Fig. 2 ▸(*a*) (convex sample), the incident angles are higher for negative *X* values and lower for positive. The situation is the opposite for the concave sample [Fig. 2 ▸(*b*)]. For a given position *X* the local incident angle reads
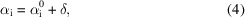
with δ = *X*/*R*. The maximal shift |δ_max_| = *l*/(2|*R*|) of the incident angle occurs at the edges of the sample.

If there exists a point where an incoming beam is tangent to the surface of the sample (point *X*_s_ in Fig. 2 ▸), a part of the sample surface is in the shadow (grey line in Fig. 2 ▸). As follows from simple geometrical considerations, the coordinate of this point is given by 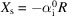. The condition for a shaded area at a nominal incident angle 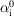 is
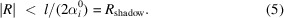
The shadow caused by curvature appears for high curvatures and small nominal incident angles. For 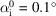 and *l* = 10 mm the shadow would appear for |*R*| < 2.86 m. This limit increases linearly with the sample length and thus for a 100 mm-long sample would be equal to 28.65 m. Only the points on the sample lying within the illuminated area contribute to the measured scattering signal. In the case of a convex sample the illuminated part corresponds to the range *X* ∈ [*X*_min_; *X*_s_]. Its length is 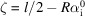, *R* < 0. For the concave sample, the illuminated area spans the range [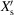, *X*_max_] [Fig. 2 ▸(*b*)], where the left limit is given by 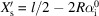
(*R* > 0), and its length is 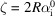 (*R* > 0). The size of the shadow (*i.e.l* − ζ) is twice as large as in the case of a convex sample. The fraction of illuminated area, ζ/*l*, is plotted as a function of |*R*| for *l* = 10 mm and for a few nominal incident angles in the supplementary material (Fig. S1).

Apart from simply yielding a distribution of incident angles, the curvature changes the density of the photon flux on the sample surface. As a consequence, the contribution of a given point on the sample to the measured scattering signal is weighted by the relative flux density: 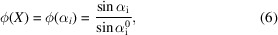
with α_i_ given by equation (4).

**Figure 3:**
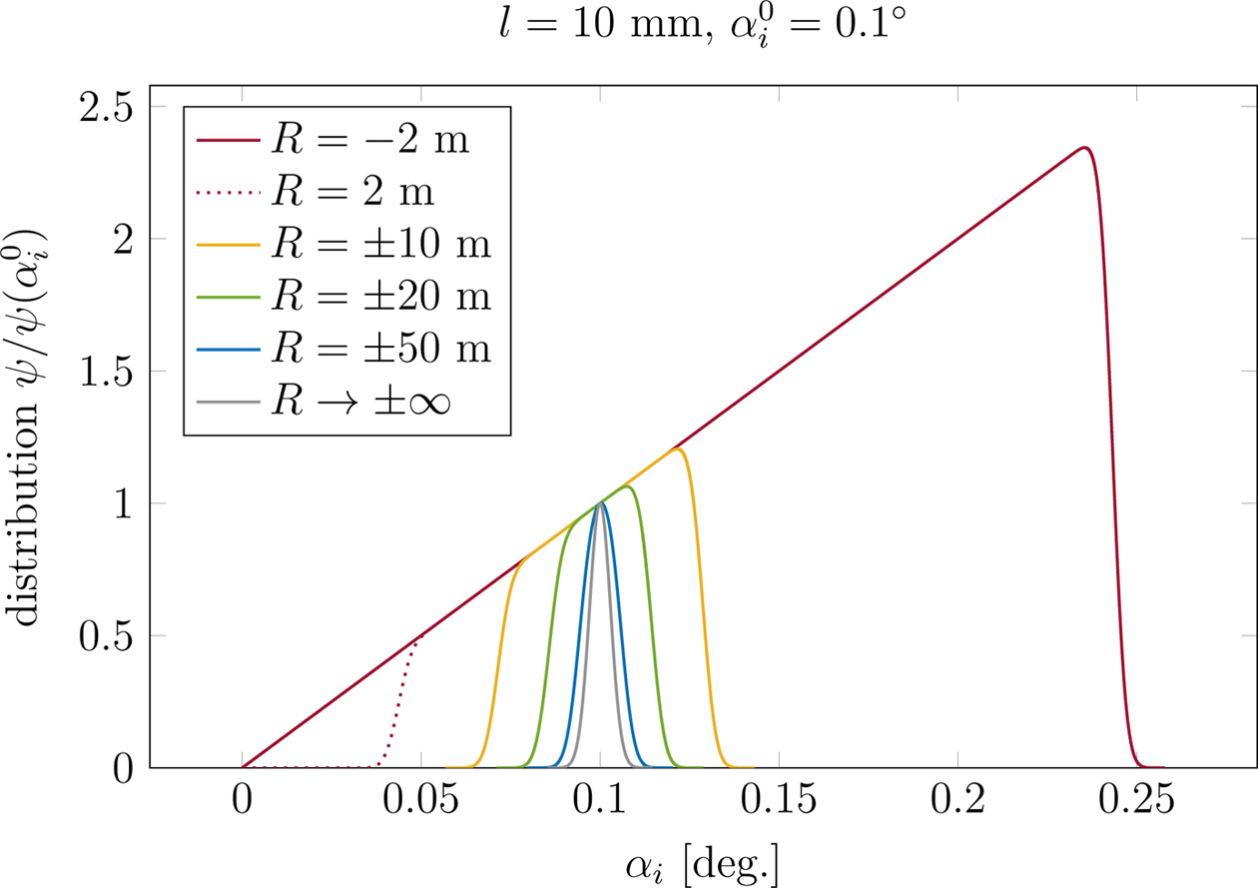
Distributions of incident angles due to curvature and divergence for *l* = 10 mm. It was assumed that the footprint of the beam in its propagation direction is larger than the sample length (ξ_*x*_ > *L*). The curves were normalized to the value at the nominal incident angle (0.1°). The difference between convex and concave samples (solid/dotted curves) is only due to the shadowing effect, in this case for |*R*| < 2.86 m. The divergence function for *R* → ±∞ is shown in grey.

So far a single **k**_i_ was considered, but in reality each X-ray beam exhibits a finite divergence, that is a distribution of **k**_i_. This results in a natural distribution of incident angles even in the case of a flat sample. Taking into account the divergence of the incoming beam, the total distribution of incident angles ψ is a convolution of the flux density distribution function [equation (6)] and divergence Θ: 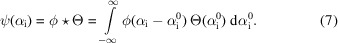
The incident angle distributions for selected *R* and *l* = 10 mm are shown in Fig. 3 ▸. To allow for comparison with our experiment presented below, for the vertical divergence a Gaussian distribution with σ_Θ_ = 120 µrad (SIXS, SOLEIL) was assumed.

Since in grazing-incidence techniques the incident angles are small, the distribution is a linear function [equation (6)] in the range 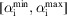 with 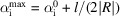. The difference between convex and concave samples is only due to the shadowing effect. For |*R*| fulfilling equation (5) there is a shadowing of the incident beam, which results in a lower limit at 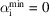 for *R* < 0 and 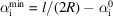 for *R* > 0 rather than 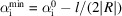 (which becomes negative). With a non-divergent beam the distribution function would fall sharply to zero at the given limit. The divergence leads to the smearing of these limits (see Fig. 3 ▸).

The divergence of the X-ray beam from a synchrotron source is usually negligible in the analysis of GISAXS data. The incident-angle distribution related to sample curvature can be compared with the distribution caused by divergence. As can be seen in Fig. 3 ▸, with decreasing |*R*| the distribution of α_i_ gets significantly broader than the divergence of the beam and its asymmetry becomes apparent. The asymmetry is caused by the change of the flux density with incident angle. For *l* = 10 mm and 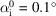 the curvature effect can be neglected for |*R*| > 50 m. For longer samples, the distribution width increases, provided that the sample is fully illuminated by the beam (ξ_*x*_ > *L*). The impact of the sample length for *R* = −100 m is shown in Fig. S2 (supplementary material). For the given curvature the width of the distribution (∼*l*/|*R*|) increases linearly with sample length (unless the curvature is large enough that the shadowing effects appear). Thus, short samples (or equivalently reduction of the beam footprint) reduce the influence of the substrate curvature on the scattering signal.

### 2.3. Exit angles

The geometry of the sample (curvature and extent along the X-ray beam propagation direction) has an impact on the exit angle. In the ideal situation of a point-like sample, each point at the detector plane receives a scattering signal related to a single exit angle. The spatial extent of the sample implies that at each point of the detector a set of scattered waves is detected, each related to a different value of exit angle (Smilgies, 2009 ▸). The curvature amplifies the significance of this phenomenon, which causes a broadening of the α_f_ range contributing to the signal measured in each detector pixel and generates a smearing of the measured signal. As discussed earlier, due to the small footprint of the beam ξ_*y*_ along the **y** direction, the impact on 2Θ_f_ is negligible.

**Figure 4:**
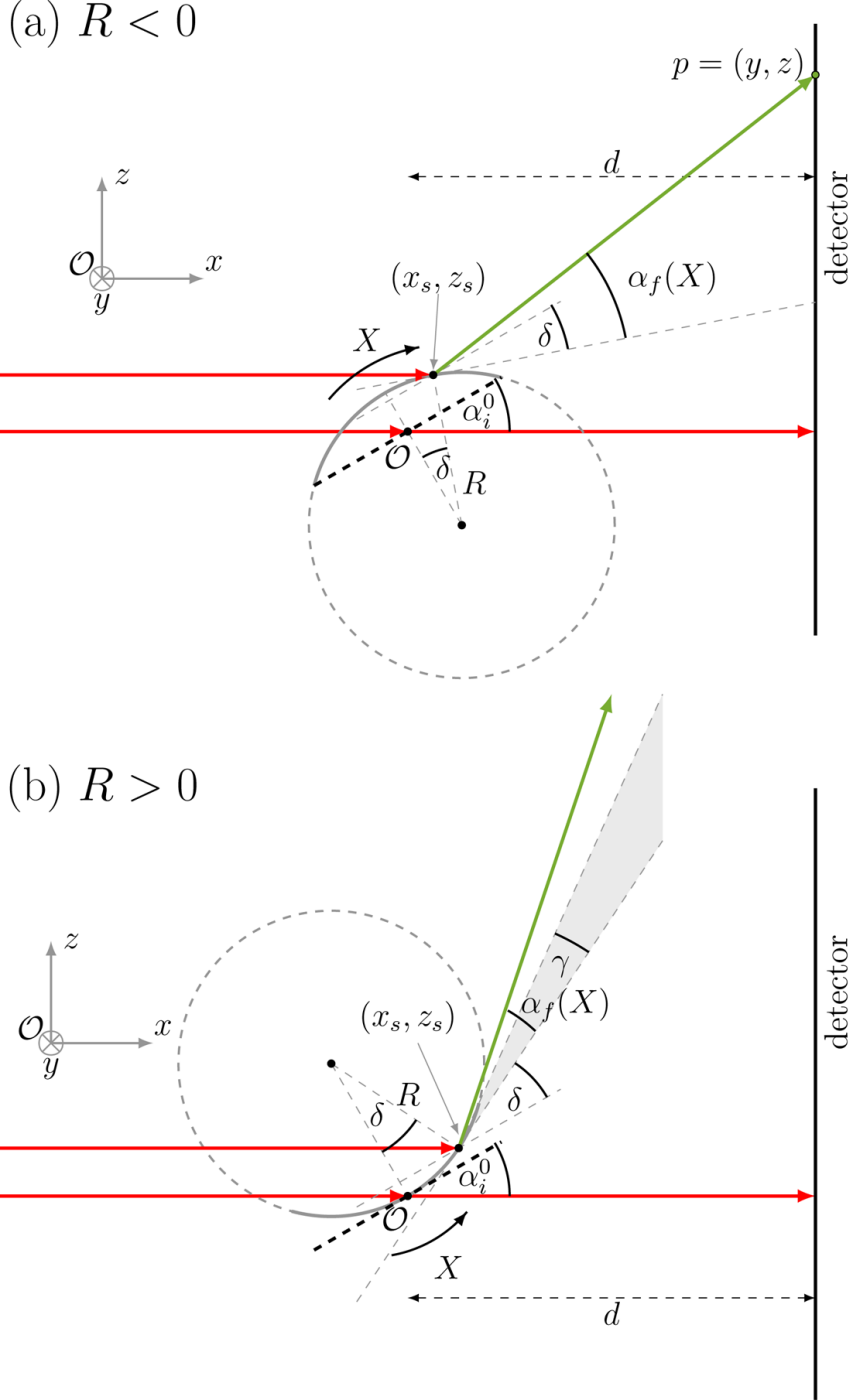
The local exit angle α_f_ for a (*a*) convex (*R* < 0) and (*b*) concave (*R* > 0) sample. Red arrows indicate the incoming beam and the green arrow represents one ray of the scattered signal. The dashed black line symbolizes the flat sample and the curved sample is drawn as a grey solid line. The local scale of the exit angles α_f_ is defined with respect to the tangent at the given point *X*. The coordinate system used for parametrization of the sample surface coordinates with respect to the detector is indicated; its origin is at the centre of the flat sample (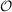). In the case of a concave sample, there is always a fraction of the signal (α_f_ < γ) that does not reach the detector. It is marked in grey in (*b*).

The local α_f_ at the curved sample is presented in Fig. 4 ▸ for both the convex and concave samples. The Cartesian coordinates (*x*_s_, *z*_s_) of the sample surface are given by the relations 
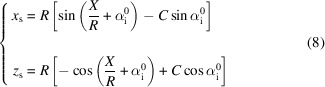
in the laboratory coordinate system depicted in Fig. 4 ▸, whose origin coincides with the centre of the flat sample. 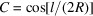 for a convex sample and *C* = 1 for a concave sample. In the limit of |*R*| → ∞ (flat sample), the relations reduce to 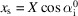, 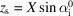.

If the extent of the sample is neglected (the sample reduced to a point at the origin 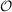 of the coordinate system, Fig. 4 ▸), the exit angles are defined as
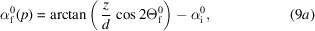

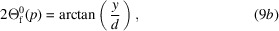
for each position *p* = (*y*, *z*) on the detector located at a distance *x* = *d* from the sample.

When the sample geometry is taken into account [equation (8)], the exit angles become
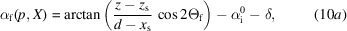

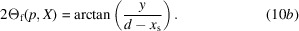
For a convex sample [Fig. 4 ▸(*a*)] all scattered waves reach the detector, whereas in the case of a concave sample [Fig. 4 ▸(*b*)] the scattered signal for α_f_ < γ does not reach the detector. The angle γ is given by 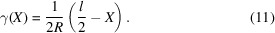
To illustrate this, we consider the shadowing effect for the sample edges. For *X* = *l*/2 the entire signal reaches the detector, while for the other edge of the sample (*X* = −*l*/2) the shadowing is maximal with γ_max_ = *l*/(2*R*). It is proportional to the sample length and increases with curvature. The dependence of γ_max_ on *R* is plotted for selected sample lengths in Fig. S3.

### 2.4. Simulation of GISAXS pattern

The GISAXS signal depends on the incident and exit angles, which are small and close to the critical angle of the substrate. In this angular region, the Fresnel coefficients vary strongly, so even a small variation in α_i_ and α_f_ can potentially impose a large change in the scattering signal. In Section 2.2 it was demonstrated how the curvature influences the local incident angle and in Section 2.3 how the sample geometry changes the range of scattered waves reaching a given point on the detector (impact of local α_f_). This mathematical approach allows for numerical calculation of the GISAXS signal taking into account the extent of the sample and its curvature.

The numerical simulations of the GISAXS pattern were performed by calculating the intensity for several positions on the sample surface *X_j_* and summing the local scattering signals. The intensity is calculated within the DWBA [equation (1)]. The curvature only affects the Fresnel coefficients if the radius of curvature is of the order of tens of wavelengths of the considered radiation (Hentschel & Schomerus, 2002 ▸; Luhn & Hentschel, 2020 ▸). Therefore here the standard form of Fresnel coefficients can be used.

In order to calculate the GISAXS signal generated by the given point *X*_*j*_, the following aspects have to be taken into account: (1) the local incident angle [equation (4)]; (2) the shadowing of the incoming beam; (3) the local exit angles α_f_ and 2Θ_f_ [equations (10)]; (4) the shadowing of the scattered signal in the case of the concave samples.

The contribution of each point *X* to the detected signal is weighted by the distribution of the photon flux on the sample surface ϕ(*X*) [equation ( 6)]. Final the GISAXS intensity reads
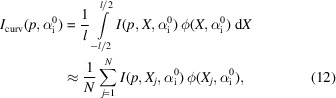
where *p* = (*y*, *z*) is the position on the detector (see Fig. 4 ▸). *N* is the number of points on the sample surface and *l* = (*N* − 1)Δ*X*. The dependence of local intensity *I* on *X* and 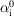 is implicit via the local incident and exit angles, such that
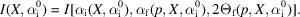
Divergence is taken into account by a numerical convolution of *I*_curv_ with the divergence function Θ, that is by repeating the calculation described above for several incident angles 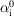. The final pattern is given by 
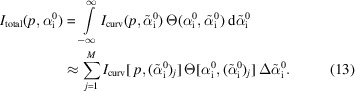
The divergence function is probed at *M* positions with angular step 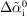. 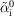 is the integration variable, and thus 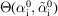 denotes the divergence distribution centred around 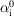. The appropriate number of sampling points can be determined by monitoring the changes in simulated function when varying *N* and *M*. In our case, *N* = 50 suffices to achieve a difference smaller than 1% with respect to *N* = 10^4^. The number of sampling points for the divergence is smaller and equals *M* = 10.

## 3. Numerical results

In the following part, the theoretical framework established in the previous section is employed to analyse the effect of sample geometry on the GISAXS pattern. For all GISAXS simulations presented here, the X-ray energy is 15 keV and the nominal incident angle is 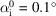. A simple model system of a single Ag cylinder on a perfectly smooth Si wafer with radius and height equal to 10 nm is used, which corresponds to the dilute case (Renaud *et al.*, 2009 ▸).

### 3.1. Effect of the sample length

**Figure 5:**
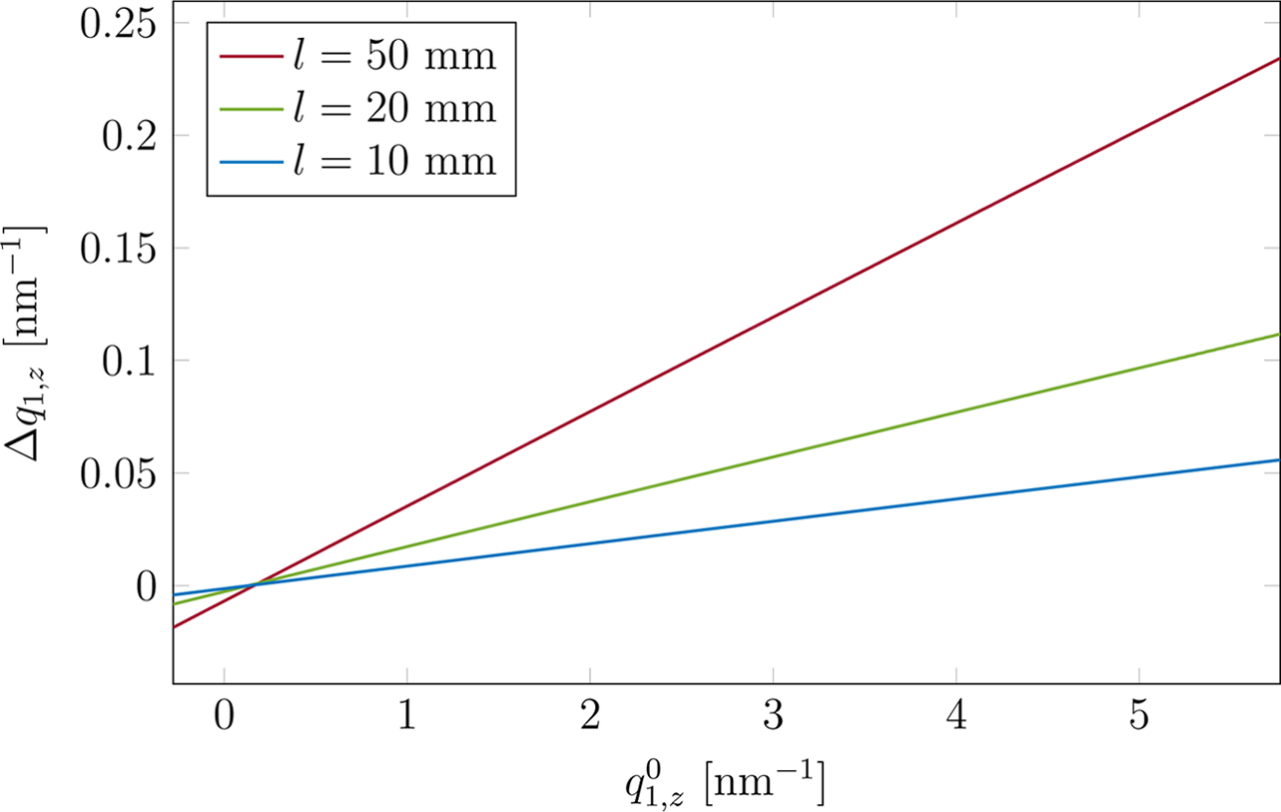
The difference in *q*_1,*z*_ calculated for the edges of the sample (*X* = ±*l*/2), Δ*q*_1,*z*_, plotted against the standard 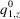 (for a point-like sample). The data correspond to 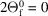 and 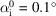 and were plotted for a few sample lengths for *d* = 1 m.

As discussed earlier, for a point-like sample each point on the detector receives a scattered wave dependent on the incident and exit angles α_i_, α_f_ and 2Θ_f_ [equations (1) and (3)]. If the spatial extent is considered, at a given detector point arrives a set of waves, coming from all points on the sample. Each wave is characterized by different α_f_ and 2Θ_f_ values, so the signal measured at a given pixel corresponds to a range of exit angles. The size of this range, or equivalently the range of **q**(α_f_, 2Θ_f_) where α_f_ and 2Θ_f_ are given by equations (10), can be used to assess the significance of the effect of sample extent. The impact of *l* is illustrated in Fig. 5 ▸ in the form of Δ*q*_1,*z*_ = *q*_1,*z*_(*l*/2) − *q*_1,*z*_(−*l*/2). This is the difference in *q*_1,*z*_ calculated for the edges of the sample (*X* = ±*l*/2), for each vertical detector pixel at 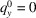 (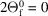), where 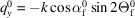. The distance from the sample to the detector is *d* = 1 m. The horizontal axis represents the *z* component of the scattering vector for the point-like sample, 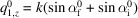. Note that this representation is independent of the specific sample (nano-objects, substrate) and thus universally shows the impact of the sample geometry. Additionally, 2D maps of the same difference in α_f_, 2Θ_f_ and **q**-vector components are shown in the supplementary materials (Figs. S4–S6). Similarly to the 1D plot, they allow us to assess the impact of the geometry of the flat sample on the measured signal, in a model-independent manner.

**Figure 6:**
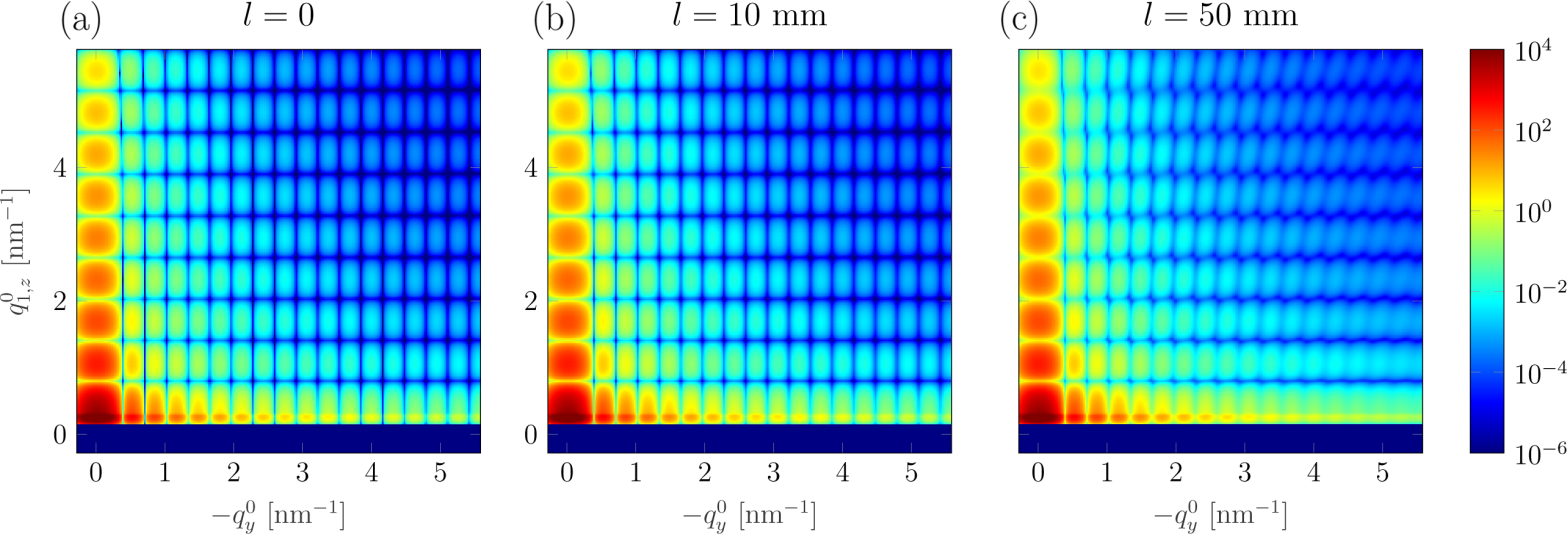
Simulated GISAXS patterns with *R* → ∞ and *d* = 1 m for a point-like sample (*a*) and taking into account the sample extent: (*b*) *l* = 10 mm, (*c*) *l* = 50 mm. For the parameters used for simulations, see the text. 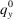 and 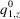 are the standard **q**-vector components for a point-like sample. Owing to the used coordinate system, 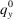 is negative for this part of the detector.

In the case of *l* = 50 mm, Δ*q*_1,*z*_ relative to 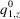 is about 5%, whereas for *l* = 10 mm it decreases almost by an order of magnitude. This has a consequence for the GISAXS pattern. In Fig. 6 ▸ the GISAXS patterns simulated by taking into account an extent of the flat sample of (*a*) *l* = 10 mm and (*b*) *l* = 50 mm are compared with the ideal case of a point-like sample (*l* = 0). The use of long samples leads to a visible distortion of the GISAXS pattern in the region of high *q*. Close to the origin of the reciprocal space, the difference between the patterns shown in Fig. 6 ▸ is negligible, due to the limited increase of Δ*q*_1,*z*_ (Fig. 5 ▸) and Δ*q*_*y*_ (Fig. S5), which increase linearly with 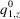 and 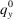, respectively. This means that the features in the GISAXS pattern at low *q* values can be directly used for structural analysis. To accurately analyse the features appearing at higher *q* values, the extent of the sample should be taken into account. From Fig. 6 ▸ one sees that for *l* = 10 mm the distortion of the pattern is not observable. Since the size of the effect scales with the sample length, it is thus generally recommended to use as short a sample as possible. Otherwise, the distortion of the pattern needs to be taken into account in the analysis.

### 3.2. Effect of substrate curvature

**Figure 7:**
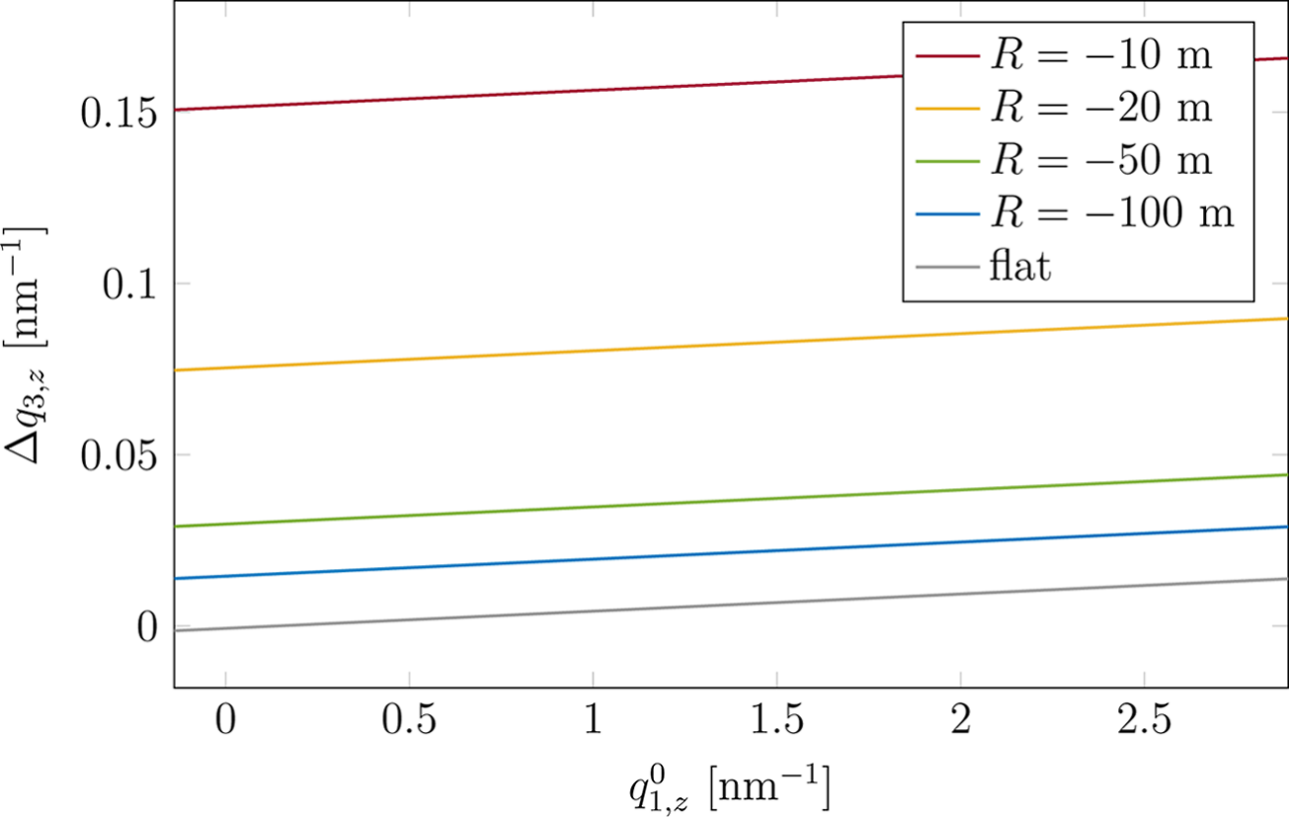
Δ*q*_3,*z*_ plotted against 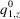 (for a point-like sample). The values for a few radii of curvature are compared. The data correspond to 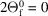, 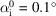 and *d* = 2 m.

In the following, the effect of substrate curvature on the GISAXS pattern is presented. For the simulations we choose *l* = 10 mm and *d* = 2 m, so that the impact of the size effect on the results is minimal. As presented in Section 2, the curvature influences not only the range of scattered waves reaching a given point on the detector [equations (10)] but also the incident angle [equation (4)]. This translates to the variation in scattering vector coordinates. The 2D maps of Δ*q*_*x*_, Δ*q*_*y*_, Δ*q*_1,*z*_ and Δ*q*_3,*z*_ are presented in the supplementary material for *R* = −10 m (Fig. S7). The impact of the curvature is the largest for *q*_2,*z*_ and *q*_3,*z*_ = −*q*_2,*z*_. This is because for a given position on the sample the incident angle [equation (4)] changes in the opposite direction to the exit angle α_f_ [equation (10*a*)] from the 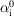 and 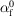 values, respectively. The curvature effect on *q*_1,*z*_ and *q*_4,*z*_ = −*q*_1,*z*_ is negligible, as the effects on incident and exit angles almost cancel out. In Fig. 7 ▸, we consider the 1D plots for Δ*q*_3,*z*_ = *q*_3,*z*_(*l*/2) − *q*_3,*z*_(−*l*/2) at 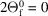 for several radii of curvature. These are compared with Δ*q*_3,*z*_ for the flat sample (grey line in Fig. 7 ▸). For *R* = −100 m, Δ*q*_3,*z*_ is approximately 2.5 times higher than for the flat sample, for *R* = −50 m the ratio is around 4, and it is already more than 10 for *R* = −20 m.

**Figure 8:**
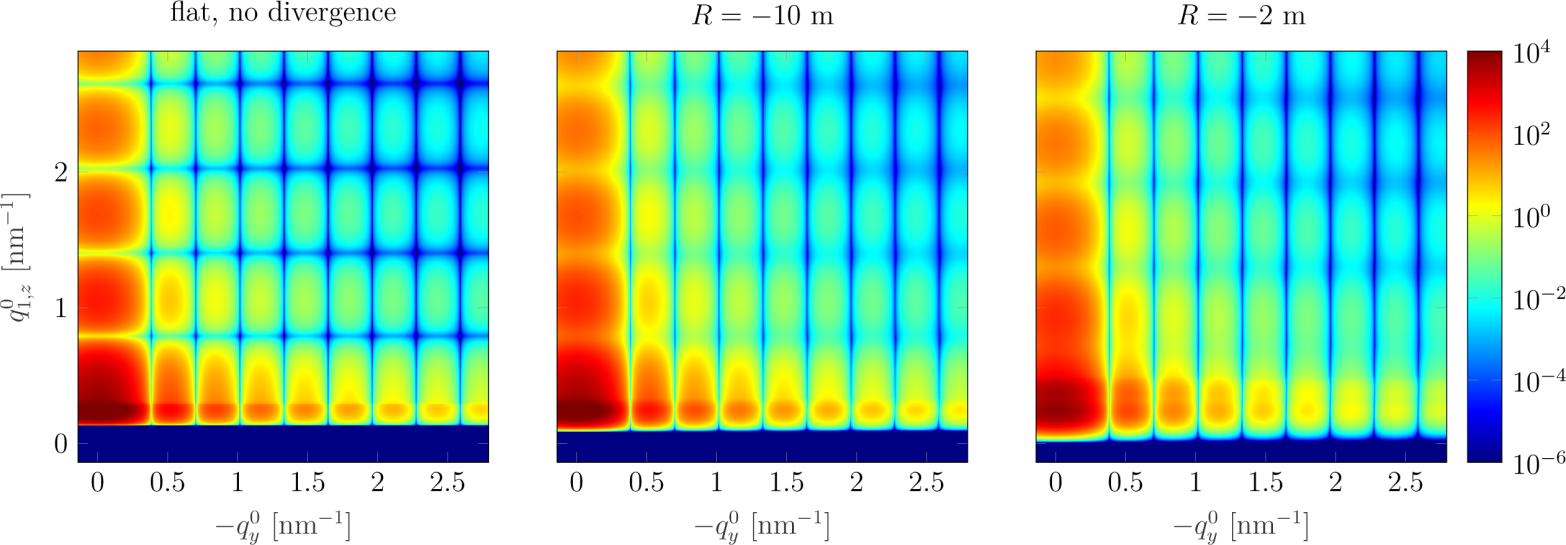
GISAXS patterns simulated for the curved convex samples for *R* = −10 m (centre) and *R* = −2 m (right). Divergence was taken into account. For comparison the pattern for a flat sample without divergence (ideal case) is shown on the left. The difference between the patterns is mostly a smearing in the vertical direction. Calculations were performed for *d* = 2 m.

Patterns simulated taking into account the substrate curvature and the divergence of the incoming beam are presented in Fig. 8 ▸. The curvature leads to the smearing of the pattern, mostly in the vertical direction. The lack of change in the horizontal direction is due to the insignificance of the curvature effect in the direction perpendicular to the incident beam for a small footprint ξ_*y*_.

**Figure 9:**
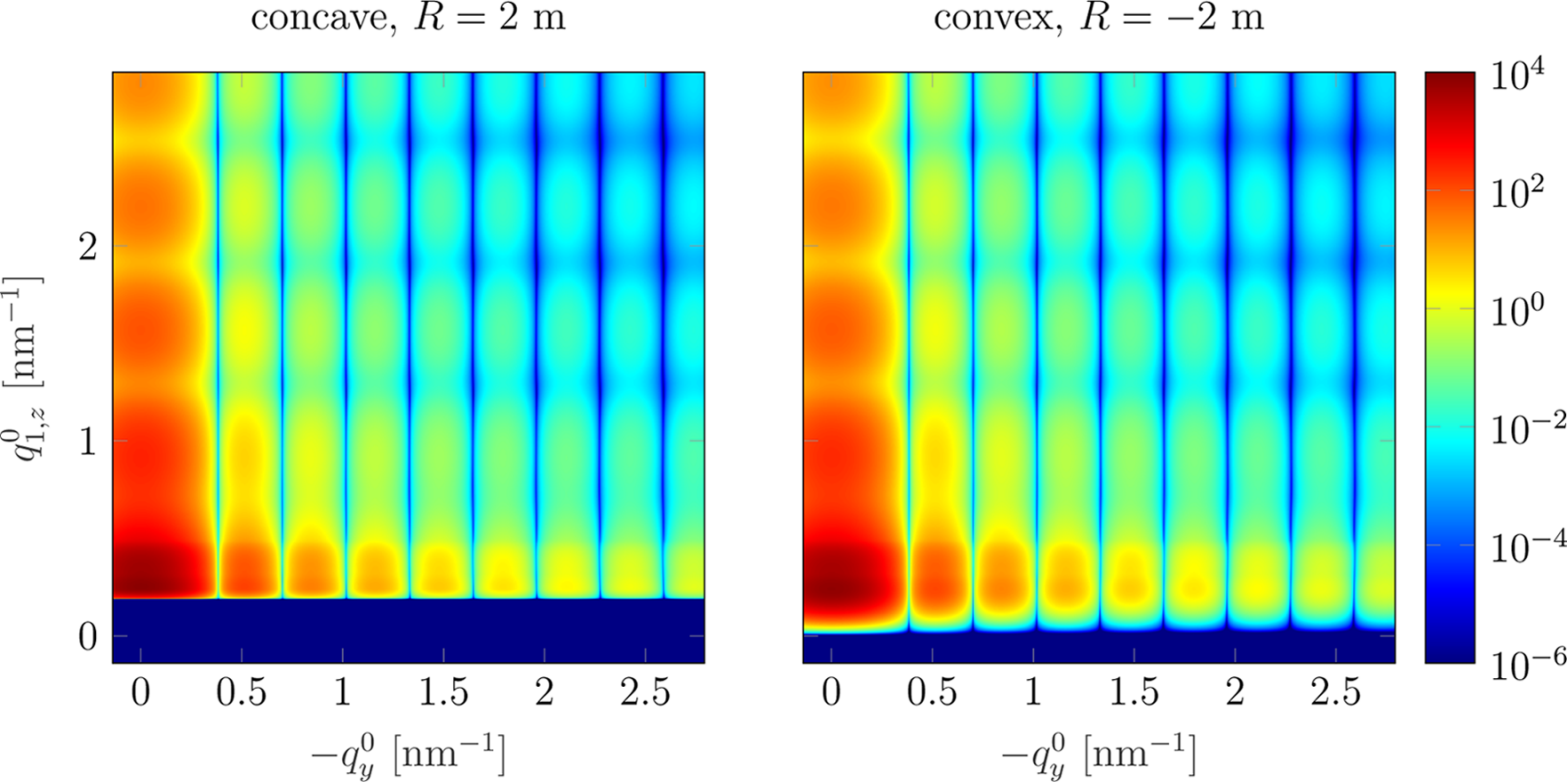
Comparison between the GISAXS patterns for the concave (left) and convex (right) samples, |*R*| = 2 m. The patterns differ mainly at small 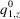, which is related to the shadowing of the scattered signal by the concave sample. Calculations were performed for *d* = 2 m.

Fig. 9 ▸ compares the patterns calculated for the concave (left) and convex (right) samples with |*R*| = 2 m. The most visible difference is shadowing of the signal for the low exit angles α_f_ (low part of the detector) in the case of the concave sample.

**Figure 10:**
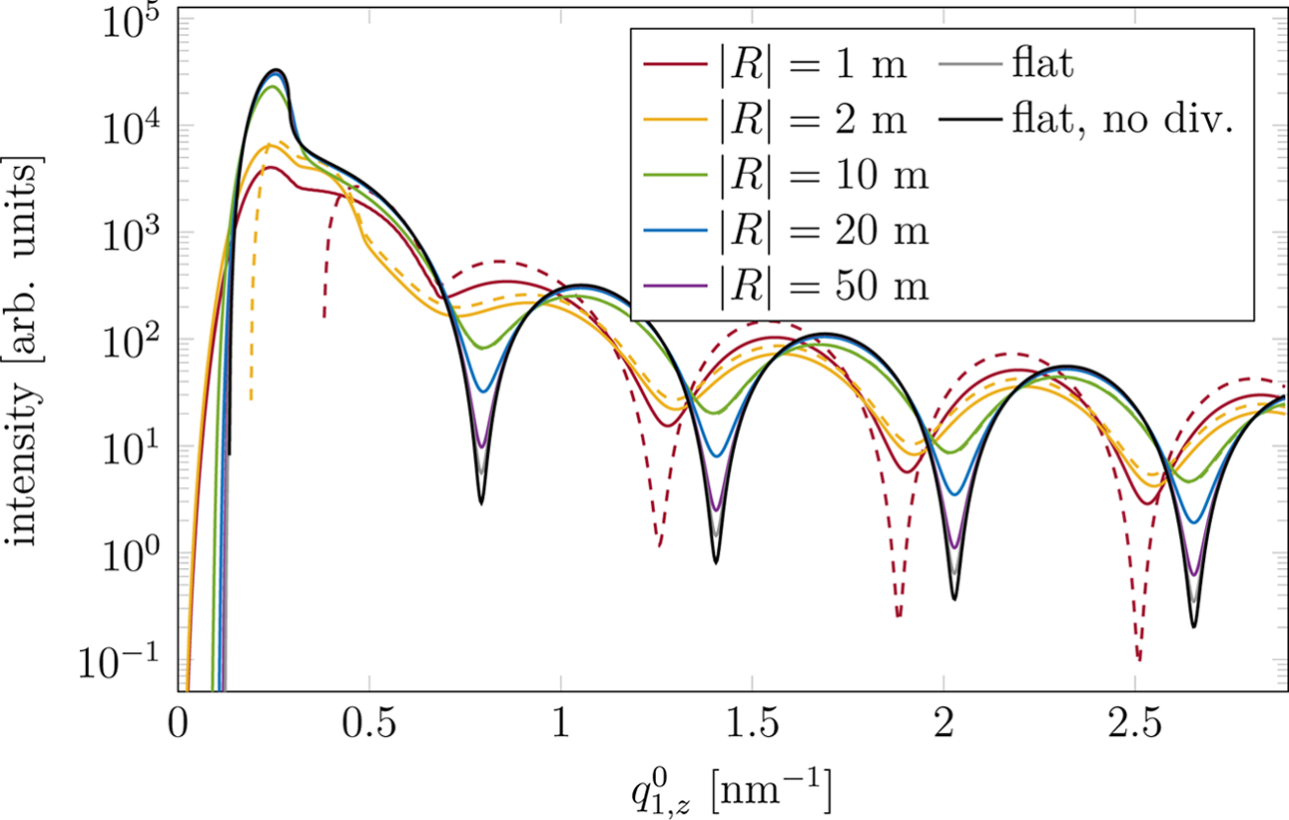
Vertical cuts through the GISAXS patterns at the position of the direct beam. Comparison for different radii of curvature as well as convex (solid lines) and concave (dashed) samples. The difference between the kind of curvature is visible only for small absolute curvature radii.

Vertical cuts through the 2D GISAXS patterns at 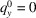 are shown in Fig. 10 ▸ for several radii of curvature and both the convex (solid lines) and concave (dashed) samples. The difference between the two shapes is visible only for small |*R*| and particularly below the limit (here |*R*_shadow_| = 2.86 m) when the shadowing of the incoming beam starts to appear. The cuts illustrate that for large |*R*| the impact of curvature is marginal and can be neglected in analysis of the GISAXS pattern. For 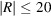
 m the smearing effect is significant. The local change of the flux on the curved sample [equation (6)] causes a shift of the maxima and minima of scattered intensity towards lower values of 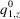. This gradual shift with decreasing |*R*| is visible in Fig. 10 ▸ for |*R*| > 2.86 m (more pronounced for high 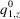). Below this limit the shadowing of the incident beam appears [equation (5)]. The regions of small α_i_ and high α_f_ stop contributing to the measured signal, which causes an immediate shift of the pattern. The smearing effect is weaker as fewer points on the sample contribute to the scattering pattern. In the case of a concave sample the size of the shaded area is twice as large as that on a convex sample with the same curvature (Fig. 2 ▸), so the shift is higher and smearing even smaller (dashed lines in Fig. 10 ▸). The other difference between the concave and convex samples is shadowing of the scattered signal by the former [Fig. 4 ▸ and equation (11)]. This effect appears for any radius of curvature but is more visible for small |*R*|. It is the reason for the difference between the curves for |*R*| = 1 m and |*R*| = 2 m in the region of small 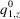.

The incident angle has a strong impact on the GISAXS signal via the reflectivity coefficients in equation (1) and through shadowing of the signal by the sample horizon. Different incident angles result in shifts of maxima and minima of the measured signal. With increasing substrate curvature, this shift is suppressed (see Fig. S8 in the supplementary material), thus reducing the impact of possible alignment errors (systematic errors of 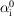). Moreover, the increase of the incident angle reduces the significance of the curvature effect on the GISAXS signal. This is presented in Fig. S9 for 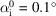 (*a*) and 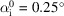 (*b*). For 0.1° the differences in vertical cuts (at 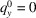) are large throughout the entire 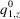 range. The situation is significantly different for 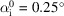. Although they are large for small 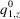 (in the vicinity of the Yoneda peak and below), the differences between the cuts for the flat sample and for *R* = ±2 m are rather moderate for higher 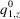 (except smearing). This is related to the fact that 0.25° is far away from the value of the critical angle for Si (∼0.119°). The differences for small 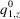 remain large because of the strong shadowing effects appearing for small |*R*|. The main conclusion is that the impact of curvature can be reduced by using a larger nominal incident angle.

**Figure 11:**
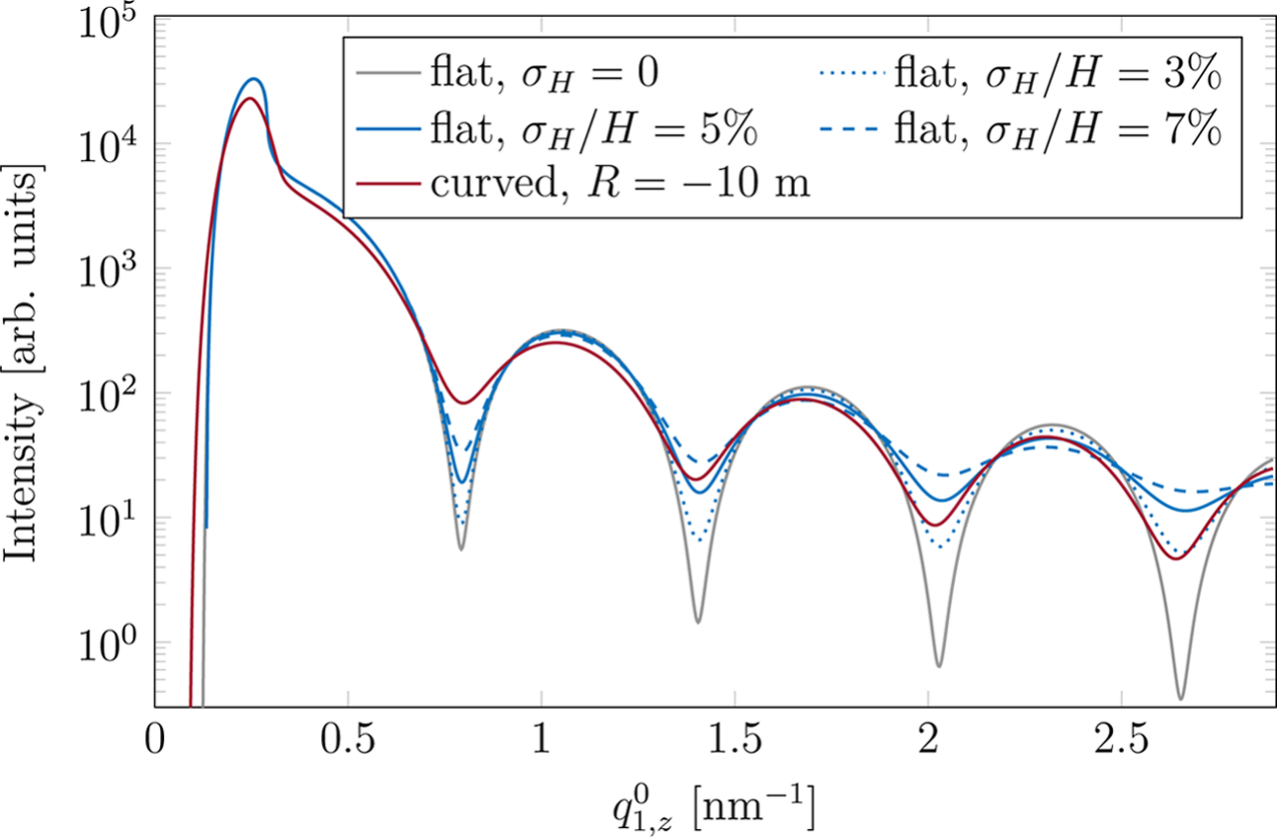
Comparison between the vertical cuts calculated for the curved sample (*R* = −10 m, red) and those for the flat sample of the Ag cylinders with height given by a Gaussian distribution with width parameter σ_*H*_ (blue). The curves for three values of σ_*H*_ are shown. For comparison, a cut calculated for the flat sample with cylinders of fixed height is shown in grey.

From the comparison of the cuts (Fig. 10 ▸) one can see that the effect of curvature manifests itself mainly as a smearing of the pattern along 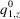 (maxima and minima become less pronounced), until the limit when the shadowing of the incident beam appears. Therefore, the effect can be compared with the effect of size distribution of nano-objects in the vertical direction. For this we calculated the GISAXS patterns for Ag cylinders (*H* = 10 nm) with a height given by a Gaussian distribution with the width parameter σ_*H*_. The calculated intensity at 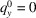 is compared with the vertical cut for the curved sample (*R* = −10 m) in Fig. 11 ▸. Both the height distribution and curvature lead to the smearing of the scattering pattern. As shown in Fig. 10 ▸, curvature additionally leads to a shift of the function extrema towards smaller 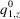, which is caused by the local change of the photon flux density. This effect cannot be reproduced by Gaussian height distribution which causes no shift. Nevertheless, comparison between the size distribution of nano-objects and the curvature effect allows for easy assessment of the size of the latter by comparison of smearing effects. The best agreement between the intensity for the curved sample and the intensity calculated with the distribution of height of the nano-cylinders is obtained for the width parameter of 0.5 nm (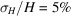). The curves for σ_*H*_ = 0.3 nm (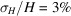) and σ_*H*_ = 0.7 nm (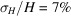) are also shown for comparison. In these cases, if the agreement is better in the low/high 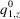 region, it becomes worse for high/low values of momentum transfer. For such a large curvature it becomes apparent that the effects of curvature and size distribution are intrinsically different and thus manifest themselves in a slightly different way in scattering patterns. Focusing only on smearing, one can see the effect of curvature for |*R*| = 10 m as similar in impact on the GISAXS pattern to the distribution of nano-object height with the relative width σ_*H*_/*H* of 5%. For |*R*| = 100 m the width of the distribution which leads to the best reproduction of the curvature effect equals 0.05 nm, or in relative terms is equal to 0.5%. Thus, for such and greater |*R*| the curvature effect can be neglected.

## 4. Experimental results

The conclusions drawn from the theoretical considerations and simulations are supported by experimental results. A real-time *in situ* experiment was performed during the deposition of a polycrystalline Ag film by magnetron sputtering on a 100 µm-thick Si substrate. GISAXS data were collected, and the curvature of the substrate was monitored using an optical measurement system (Fillon *et al.*, 2010*a* ▸; Abadias *et al.*, 2015 ▸; Abadias *et al.*, 2018 ▸). The experiment provided a set of GISAXS patterns recorded for a sample whose curvature evolved during Ag deposition.

### 4.1. Experimental details

**Figure 12:**
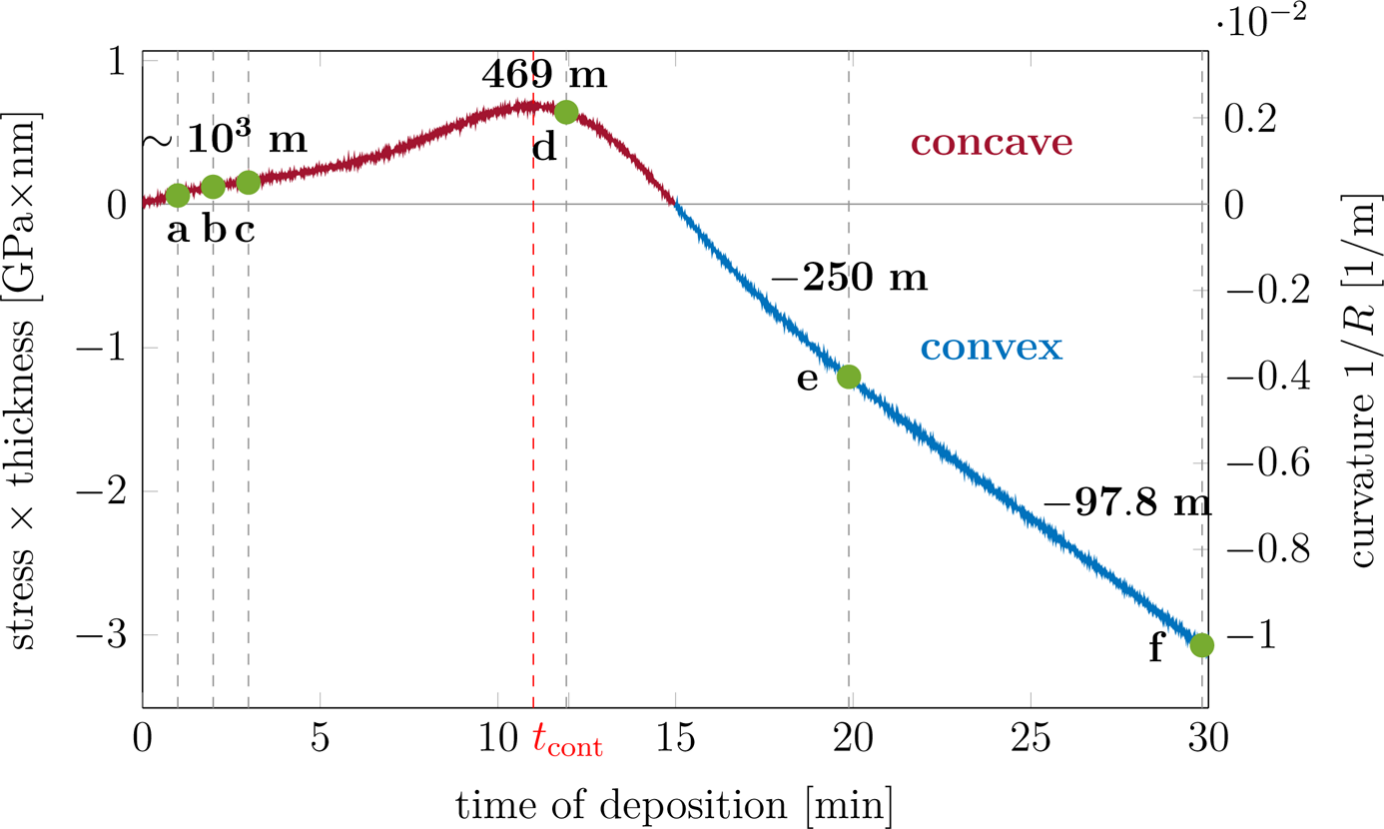
Curvature and stress data recorded during the deposition of the Ag layer. Green points and dashed vertical lines mark the times for which GISAXS data were analysed; *a*–*f* indicate the corresponding experimental GISAXS patterns shown in Fig. 13 ▸. Above the points, corresponding *R* values of the substrate at the given moment are indicated.

The experimental results presented here were obtained during synchrotron beamtime at the beamline SIXS, syn­chrotron SOLEIL (France). The incident X-ray beam had an energy of 15 keV and a size of 0.06 × 0.15 mm^2^ (vertical × horizontal). The nominal incident angle 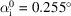 was close to the critical angle of Ag (0.234°), enhancing the contribution from the surface layer. Additionally, a sufficiently high incident angle reduces the potential impact of the curvature, as discussed in Section 3.2 [see Fig. S9(*b*)]. A 2D detector (Eiger 1M, DECTRIS AG, Switzerland) was positioned at the distance *d* = 2.459 m to the sample. A portable sputtering chamber (Krause *et al.*, 2012 ▸) was installed on a hexapod on the multi-environment diffractometer. The Ag layer was deposited on a thin (naturally surface-oxidized) Si substrate with dimensions of 13 × 11 × 0.1 mm^3^, which is free to bend and allows for real-time curvature/stress monitoring. The film was deposited via DC magnetron sputtering at room temperature in Ar plasma with a small addition of gaseous N_2_ (10%), which allows for improved wetting of the Ag layers and obtaining a conductive layer at sufficiently low thickness (Sarakinos *et al.*, 2024 ▸; Zapata *et al.*, 2024 ▸). Moreover, the growth in mixed Ar/N_2_ plasma results in faster development of the compressive stress in the continuous film (blue region in Fig. 12) compared with the growth in pure Ar (Sarakinos *et al.*, 2024 ▸). The distance from the Ag sputter target to the substrate was 325 mm and the magnetron power was 20 W. The deposition lasted 30 min. A GISAXS pattern was recorded every second during the deposition. The curvature of the sample during deposition was monitored with a multiple-beam optical stress sensor (kSA MOS, k-Space Associates Inc., USA) (Fillon *et al.*, 2010*a* ▸; Abadias *et al.*, 2015 ▸). A pattern of nine spots served for determining the curvature. The Stoney (1909 ▸) equation was used to compute the film force per unit width, which is equivalent to the product of stress × thickness. By convention, positive (negative) values correspond to tensile (compressive) stress.

### 4.2. Results

The simultaneous evolution of the GISAXS pattern and the stress is presented in the video file available in the supplementary material. The curvature/stress curve is shown in Fig. 12 ▸. Three regions are clearly visible: at first an increase in tensile stress, followed by a tensile peak whose maximum occurs at *t*_cont_ ∼ 11 min corresponding to the film continuity (Abadias *et al.*, 2015 ▸) and then a compressive regime. The concavity of the sample changes during the growth (at *t* ≅ 15 min).

**Figure 13:**
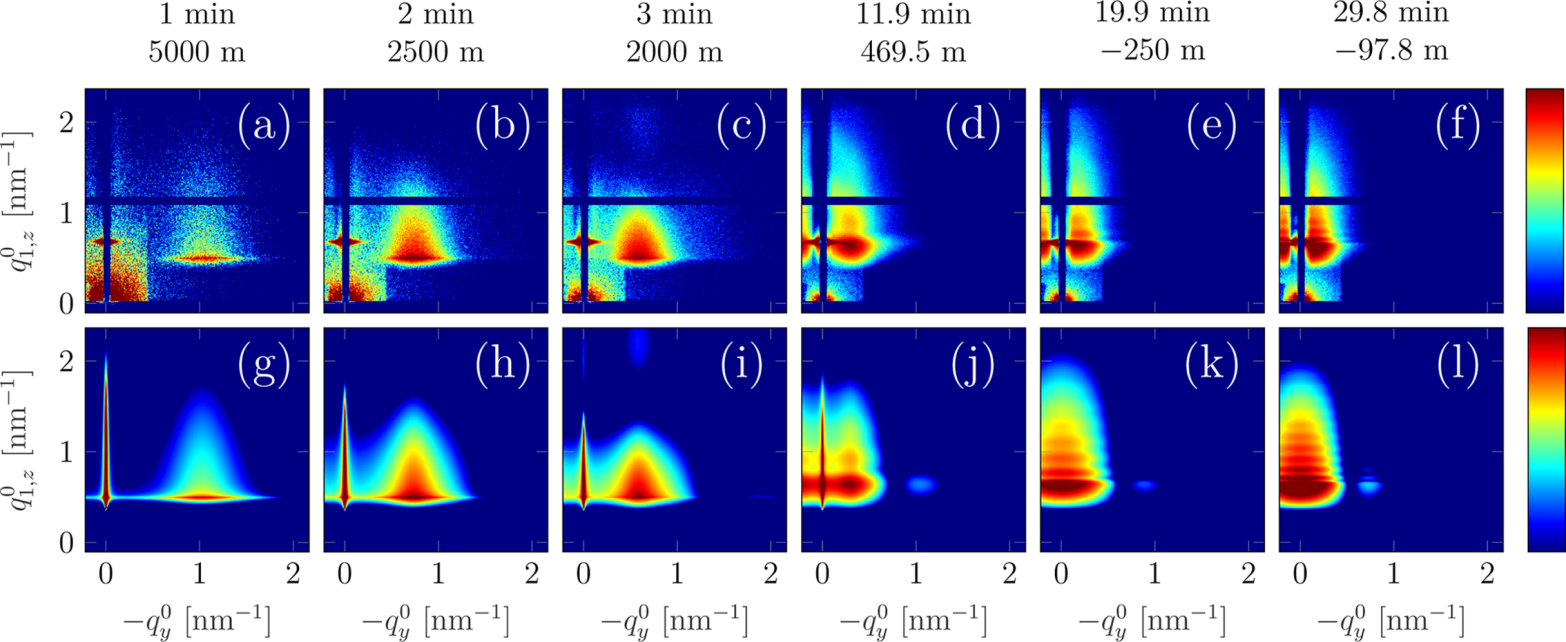
Comparison of the GISAXS data and simulated patterns for selected times during the film deposition. The upper plots (*a*–*f*) show the experimental pattern, and the simulation results are given in the second row (*g*–*l*). A good agreement can be observed in all cases. The values above the images indicate the deposition time and the corresponding substrate curvature. The intensity (colour) is plotted on a logarithmic scale.

**Figure 14:**
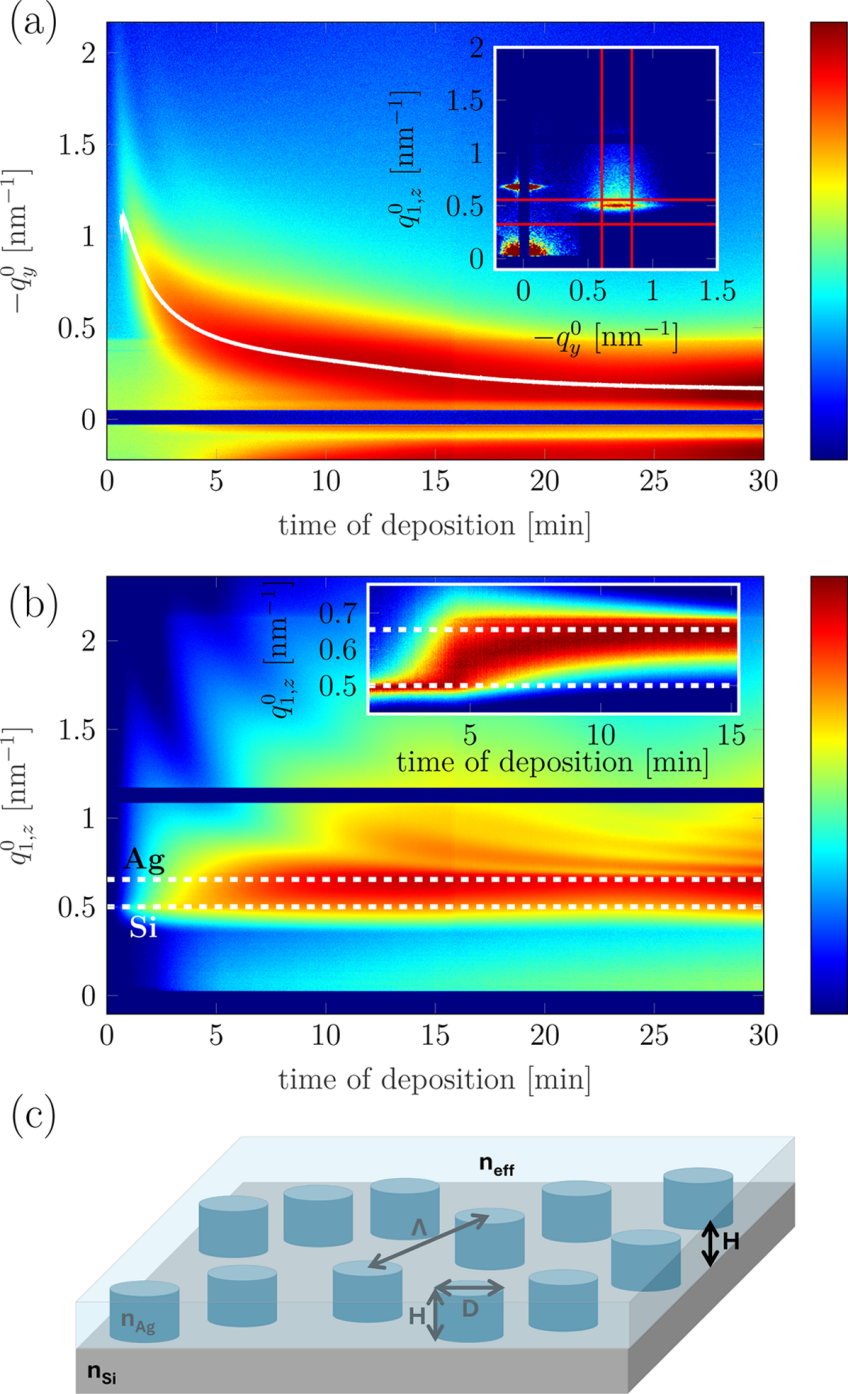
The evolution of cuts through the 2D GISAXS pattern during deposition of Ag: (*a*) horizontal cut at the Yoneda peak; (*b*) vertical cut at the interference peak. The inset in (*a*) shows an exemplary GISAXS pattern with the integration regions indicated by red lines. The inset in (*b*) shows a section of map (*b*) on a linear scale to better visualize the shift of the Yoneda peak at the beginning of the growth. The white line in (*a*) indicates the position of the interference peak determined from the fit by the Voigt function. White dashed lines in (*b*) indicate the positions in 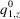 corresponding to critical angles of pure Si and pure Ag. (*c*) Structural model used for the analysis of GISAXS. For explanation of the symbols, see the text. In all cases apart from the inset in (*b*), the intensity (colour) is plotted on a logarithmic scale.

The evolution of the entire 2D GISAXS pattern is presented in the upper row of Fig. 13 ▸, which is based on selected representative patterns, and in the video in the supplementary material. The evolution is characteristic of the Volmer–Weber growth of a noble metal on a weakly interacting substrate (Lazzari *et al.*, 2007*b* ▸). At the early stages the pattern evolution is dominated by the appearance of an interference peak and then its movement towards lower 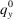 (see Figs. 13 ▸ and 14 ▸). The position of the peak in 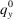 can be attributed to the average distance between the centres of the islands. Near film continuity [∼11 min, see pattern (*d*)], the two interference peaks (the second peak becomes visible in the studied 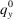 range only for later deposition stages) merge at the central rod (at 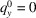). The nano-island growth in height is manifested as the appearance of higher-order oscillations in the vertical direction. The evolution of the GISAXS signal is presented in another way in Fig. 14 ▸. The colour maps show the evolution of horizontal [Fig. 14 ▸(*a*)] and vertical [Fig. 14 ▸(*b*)] cuts as a function of the deposition time. The horizontal cuts show the movement of the interference peak position towards smaller 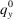 as the growth of the film progresses. This indicates the increasing island distance due to coalescence between Ag islands. The Yoneda peak is a feature visible in the vertical cuts. It appears at 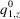 corresponding to an exit angle α_f_ equal to the critical angle of the medium and is related to the average electron density (Renaud *et al.*, 2009 ▸). The evolution of the Yoneda peak position from 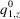 of pure Si (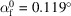) towards 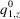 of pure Ag (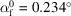) is visible in Fig. 14 ▸(*b*). The penetration depth of X-rays for the grazing angle of 0.255° is limited to a few tens of nanometres, so once sufficient Ag is deposited on the substrate, the probed average electron density is determined by Ag. Additionally a higher-order maximum is visible in the vertical cut starting from around 5 min of deposition, which indicates the vertical growth.

Selected GISAXS patterns from various stages of the deposition (*i.e.* below and above *t*_cont_) were analysed in detail (Table 1 ▸ and Fig. 13 ▸). Since at all deposition stages |*R*| > 50 m, the curvature effect was neglected in the analysis. In order to extract the parameters describing the morphology of the growing film, the GISAXS patterns were simulated and compared with the experimental data. In this way the model parameters were refined. The model used for the analysis is shown schematically in Fig. 14 ▸(*c*). The Ag nano-islands were assumed to have the shape of cylinders with in-plane diameter *D* and height *H*. A monodisperse assembly was considered and the interference function was described by the radial paracrystal model with two parameters: interparticle distance Λ and standard deviation σ_Λ_. The Ag islands were considered to be embedded in an *effective* Ag/vacuum layer with thickness *t*_eff_ = *H* and refractive index *n*_eff_ = 1 − τδ_Ag_ − *i*τβ_Ag_, where *n*_Ag_ = 1 − δ_Ag_ − *i*β_Ag_ is the refractive index of pure Ag and τ is the volume fraction of Ag in the effective layer (Fig. 14*c*). Generally, this effective interface model (Babonneau *et al.*, 2009 ▸) can be regarded as a simplification of the graded interface model (Lazzari *et al.*, 2007*a* ▸; Lazzari *et al.*, 2007*b* ▸), where a continuous variation of *n* in the growing layer is assumed. In the specific case of cylindrical islands, those two models are identical. The effective interface model allows for correct reproduction of the position and shape of the Yoneda peak, which change during the deposition due to the increasing average electron density of the layer. Above *t*_cont_, the islands were assumed to be situated at the surface of a continuous Ag layer of thickness *T*_Ag_. The data were analysed using the *FitGISAXS* software (Babonneau, 2010 ▸). The resulting model parameters for selected GISAXS patterns are collected in Table 1 ▸.

The parameters extracted from the modelling of the GISAXS data reflect the growth of islands as the deposition progresses. Particularly, the diameter of the islands increases continuously with time. The height of the cylinders increases until continuity. The interparticle distance Λ and standard deviation σ_Λ_ also increase until continuity of the film is reached, which may be attributed to the coalescence process. Then, a continuous Ag layer below the islands is assumed. Both the thickness of the layer and the diameter of the islands increase with the deposition time. The value of τ, that is the volume fraction of Ag in the layer containing Ag islands, does not change significantly until late deposition stages. This is probably related to the high density of the island network during the entire deposition.

A comparison between the GISAXS data and simulated patterns is presented in Fig. 13 ▸. A good agreement was reached in all cases, both for early deposition stages and for the continuous layer. Remarkably, also for high curvatures (*R* ≈ −100 m), at the end of deposition, the GISAXS patterns could be successfully reproduced and model-based information about the morphology of the film extracted.

## 5. Summary

In this work we addressed the effect of the sample geometry (both size and curvature) on the GISAXS signal. A thorough theoretical description of the effect was presented in Section 2. Particularly, it was shown how the curvature of the sample translates into the local deviation of the incident angle from its value for the flat sample. The shadowing of the incident X-ray beam for highly curved samples was discussed. The comparison of the total incident angle distribution with the natural α_i_ distribution caused by beam divergence (Fig. 3 ▸) implies that the curvature effect can be neglected in the case of comparable widths of those distributions. If the effective length of the sample surface illuminated by the X-ray beam is small compared with the sample-to-detector distance (typically 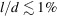), the effect of curvature is insignificant for |*R*| > 50 m.

Subsequently, the impact of sample geometry (its extent and curvature) on the range of scattered waves reaching a selected point on the detector was addressed. Both increasing sample size and increasing curvature lead to an increase of the range of scattered waves (characterized by a range of exit angles) at a given point at the detector. For convex samples the entire signal emerging from the sample reaches the detector, but in the case of a concave shape some of the scattered waves may be blocked by the sample itself. These results were then used to calculate the GISAXS signal, taking into account the geometry of the sample.

Using the information presented in Sections 2.2 and 2.3, we could simulate the full 2D GISAXS pattern taking into account the sample extent and curvature. Regarding the former, both model-independent Δ**q** plots and full GISAXS pattern simulation show that the length of the sample leads to the distortion of the scattering pattern (due to the fan of scattered waves reaching each detector point). The shape of the function Δ**q**(**q**) and its values reported by Smilgies (2009 ▸) are in agreement with our results. The higher the magnitude of the scattering vector, the larger the deviation from the ideal case of a point-like sample. Experiments with a large ratio of the sample length to *d* have been reported previously (Jeng *et al.*, 2010 ▸; Babonneau *et al.*, 2005 ▸; Jousseaume *et al.*, 2009 ▸).

Finally, we demonstrated the impact of the substrate curvature on the GISAXS pattern. We provided a plot of model-independent Δ*q*_3,*z*_ (Fig. 7 ▸) for the curved sample and compared it with Δ*q*_3,*z*_ for the flat sample. Comparison of vertical cuts through the GISAXS patterns (Fig. 10 ▸) implies that for |*R*| greater than |*R*_shadow_| [equation (5)] the curvature leads to the smearing of the pattern and a small shift of extrema towards smaller 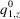 values. In the case of even smaller |*R*| the effect becomes very pronounced and the difference between convex and concave samples is visible (due to the different sizes of the shadow and shadowing of the scattered signal). A comparison between curvature and nano-object height distribution reveals that the two effects are different in nature: while both lead to intensity smearing, only curvature causes a shift of the intensity extrema. Nevertheless, the comparison of the smearing caused by the two effects allows for the estimation of the importance of curvature effects (Fig. 11 ▸).

The theoretical formalism presented here for GISAXS can be also applied to other GIXS methods, (*e.g.* XRR and grazing-incidence diffraction, which probes the crystallinity of the near-surface region), as the generic mechanism causing the potential scattering-signal changes is always the same. The formalism for XRR developed by Konovalov *et al.* (2022 ▸) is similar to ours but uses a different parametrization of the sample surface, differently describes the phenomenon of the local change of the photon flux density and does not treat the case of concave shape. Both in our formalism and in the work of Konovalov *et al.*, the curvature in the direction perpen­dicular to the incoming beam is neglected. In the latter the radii of curvature considered are substantially lower than in our case. Despite that, this assumption is valid for the presented experimental data (Konovalov *et al.*, 2022 ▸; Belova *et al.*, 2023 ▸). The impact of curvature perpendicular to the beam was treated by Briscoe *et al.* (2012 ▸). Our formalism could be extended to include this case, if the lateral size of the beam is larger or the curvature is even greater than is considered in the papers of Konovalov *et al.* (2022 ▸) and Belova *et al.* (2023 ▸).

In the *Experimental results*[Sec sec4] section, we presented the results from the real-time experiment combining GISAXS and substrate curvature measurements during the growth of an Ag thin film by magnetron sputtering in Ar/N_2_ plasma discharge. We observed an evolution of the GISAXS patterns (Fig. 14 ▸) and stress state (Fig. 12 ▸) characteristic of the Volmer–Weber growth of Ag on SiO_*x*_. The determination of the stress state in the film via substrate curvature measurements is an experimentally simple technique and readily delivers information about the microstructural evolution of the thin film. It can be used for monitoring of the impact of the deposition parameters on the growth of the thin film (Fillon *et al.*, 2010*b* ▸; Abadias *et al.*, 2018 ▸; Krause *et al.*, 2023 ▸; Sarakinos *et al.*, 2024 ▸). By combining substrate curvature measurements with real-time GISAXS one can understand which changes in morphology lead to the observed shape of the stress curve.

We successfully analysed the experimental GISAXS patterns (Fig. 13 ▸) using a standard model of monodisperse cylinders and lateral ordering described by the paracrystal function, without the need for taking into account geometrical effects (extent and curvature). The structural parameters extracted from the modelling (Table 1 ▸) reflect the lateral growth of the islands throughout the entire deposition process, especially in the early stages as a result of island coalescence. Their height increases at the early deposition stages and then does not change significantly, which is related to the onset of the film continuity. This experimental study and analysis show the feasibility of combining GISAXS with optical substrate curvature monitoring for investigation of thin-film growth.

The theoretical approach presented in this work for GISAXS can also be directly applied to a range of experimental problems. For instance, the use of GISAXS as a nano-metrology tool (Wernecke *et al.*, 2014 ▸) could be extended to the cases of long and curved samples. In case of electronic devices on very thin or flexible substrates (Kang *et al.*, 2005 ▸; Shimoto *et al.*, 2004 ▸; Vella *et al.*, 2009 ▸; Buencuerpo *et al.*, 2012 ▸; Yordanov & Angelopoulos, 2013 ▸; Rani *et al.*, 2023 ▸; Mönch *et al.*, 2011 ▸; Sun *et al.*, 2005 ▸), a rigorous assessment of the possible impact of their curvature can allow for high-quality GISAXS analysis yielding morphological parameters. GISAXS could be potentially employed here during the preparation phase or to check if the device was fabricated as desired. The same applies to devices on flexible substrates, which nowadays attract increasing interest (Lucarini *et al.*, 2021 ▸; Wei *et al.*, 2020 ▸). Taking into account curvature effects would be essential for investigations of nano-objects at the surface of droplets, which exhibit low |*R*| (Campolongo *et al.*, 2011 ▸; Sartori *et al.*, 2022 ▸; Konovalov *et al.*, 2022 ▸; Belova *et al.*, 2023 ▸).

In summary, we considered the possible influence of the sample geometry on the GISAXS signal. More specifically we addressed the impact of the sample length and its curvature. The combination of real-time substrate curvature measurements and GISAXS for studying the growth of thin films deposited by magnetron sputtering constituted the main motivation for this work. We focused on GISAXS, as this method provides morphological information, complementary to that obtained from substrate curvature measurement. Nevertheless, the presented formalism can be used for experimental study with other GIXS methods, where the geometry of the sample may influence the scattering signal.

## Supplementary Material

Additional graphs illustrating some aspects of the curvature and size effect and their influence on final angles and <b>q</b>-vector components. The dependence of the results on the nominal incidence angle is also presented. DOI: 10.1107/S1600576725010726/xx5087sup1.pdf

The simultaneous evolution of the GISAXS pattern and the stress. DOI: 10.1107/S1600576725010726/xx5087sup2.mp4

## Figures and Tables

**Table 1:** The morphological parameters of the growing Ag layer obtained from the analysis of selected GISAXS patterns recorded at the time *t* of deposition The radius of curvature of the sample at those time steps is shown in the second column.

*t* (min)	*R* (m)	*T*_Ag_ (nm)	τ	*D* (nm)	*H* (nm)	Λ (nm)	σ_Λ_ (nm)
1	5000	–	0.52	2.95	1.93	4.86	2.00
2	2500	–	0.49	4.42	3.11	7.35	2.51
3	2000	–	0.55	5.40	4.25	9.30	3.15
11.9	469.5	7	0.45	9.90	3.48	13.55	7.46
19.9	−250	14	0.46	11.65	3.01	–	–
29.8	−97.8	21	0.38	14.19	3.05	–	–

## Data Availability

The data are available upon request.
