# Supplementary material for: Impact of substrate curvature on grazing-incidence small-angle X-ray scattering signal: theory and example of Ag thin-film growth
Source: J Appl Crystallogr. 2026 Feb 1;59(Pt 1):93–107. doi: 10.1107/S1600576725010726 (PMC12871481; doi:10.1107/S1600576725010726)
Supplement: Supplementary file 1 [file j-59-00093-sup1.pdf]

JOURNAL OF APPLIED CRYSTALLOGRAPHY

Volume 59 (2026)

Supporting information for article:

Impact of substrate curvature on GISAXS signal: theory and example of Ag thin-film growth

Michał Kamiński, Bärbel Krause, Gregory Abadias, Alessandro Coati, Yves Garreau, Anny Michel, Andrea Resta, Karan Solanki, Alina Vlad and David Babonneau

1 Supplementary material

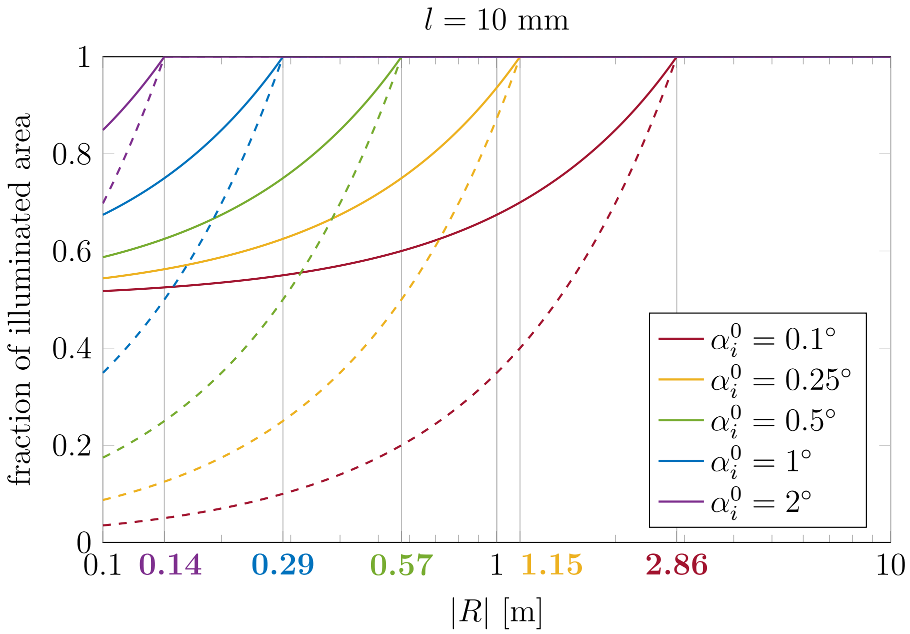

Figure S1: Fraction of the area illuminated by the incident beam ζ/l plotted as a function of the modulus of the radius of curvature (logarithmic scale) and several nominal incident angles α_i^0 ($l = 10$ mm). A shadow appears as an effect of sample curvature. The solid lines correspond to the convex sample ($R < 0$), whereas the dashed ones to the concave shape ($R > 0$). The size of the shadow ($l - \zeta$) is twice larger for the concave sample comparing to the convex one. The graph shows that the effect appears only for small radii of curvature, in the order of meters. The limiting radius $|R| = l/(2\alpha_i^0)$ is marked as bold values on the x axis for each nominal incident angle.

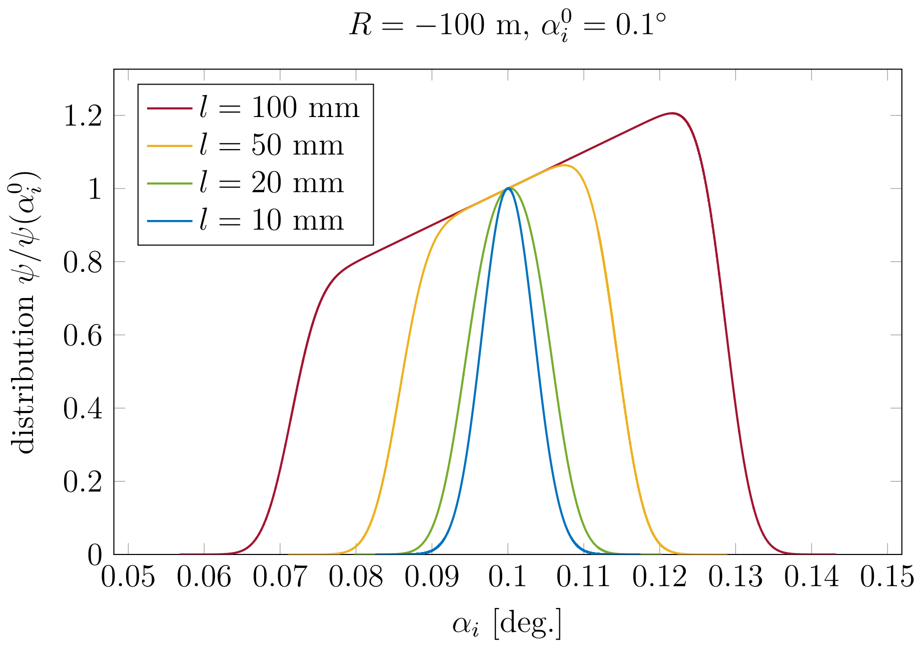

Figure S2: Dependence of the distributions of incidence angles on the length of the sample l. The radius of curvature is equal to $R = -100$ m, the nominal incidence angle $\alpha_i^0 = 0.1°$. It was assumed that the footprint of the beam in its propagation direction is larger than the sample length ($\xi_x > L$). One can notice an increasing distribution width with the sample length. Thus, one should use short samples to reduce the influence of curvature effect.

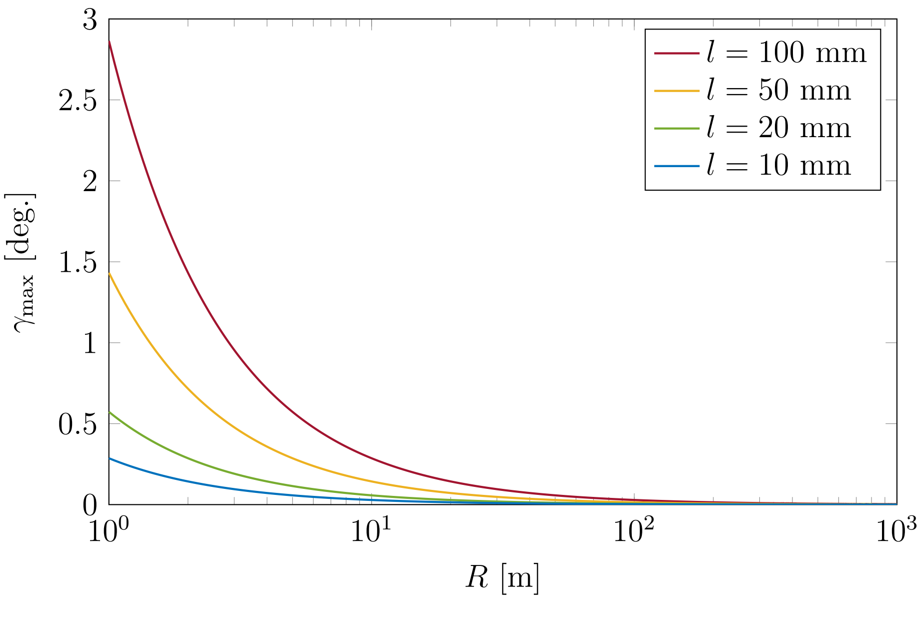

Figure S3: The dependence of the angle $\gamma_{\max}$ on the radius of curvature. The function was plotted for several sample lengths l. $\gamma_{\max}$ can be regarded as a measure of the size of the effect of shadowing of the scattered signal on the way to the detector due to the concave curvature of the substrate. One can notice that the effect scales linearly with the sample length.

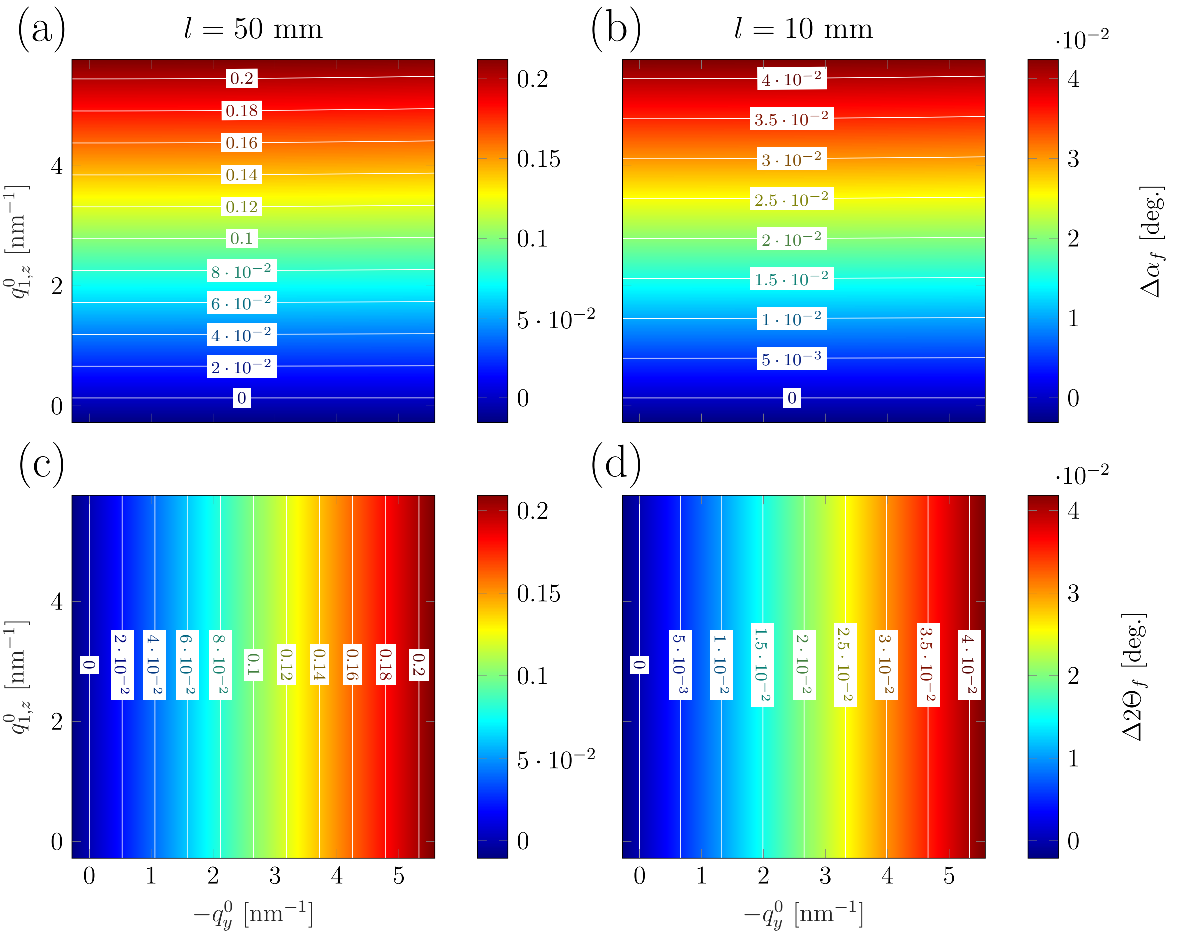

Figure S4: The difference in exit angles α_f (a,b) and $2\Theta_f$ (c,d) between the edge positions on the flat sample ($X = -l/2$ and $X = l/2$). The left graphs show the exit angles for $l = 50$ mm, whereas the right ones for $l = 10$ mm. One can observe that the change in the angles due to the sample extent is significant in the first case ($\sim 5\%$ with respect to the negligence of size) and negligible for the second setting ($\sim 1\%$). The data are calculated for $d = 1$ m and $\alpha_i^0 = 0.1°$.

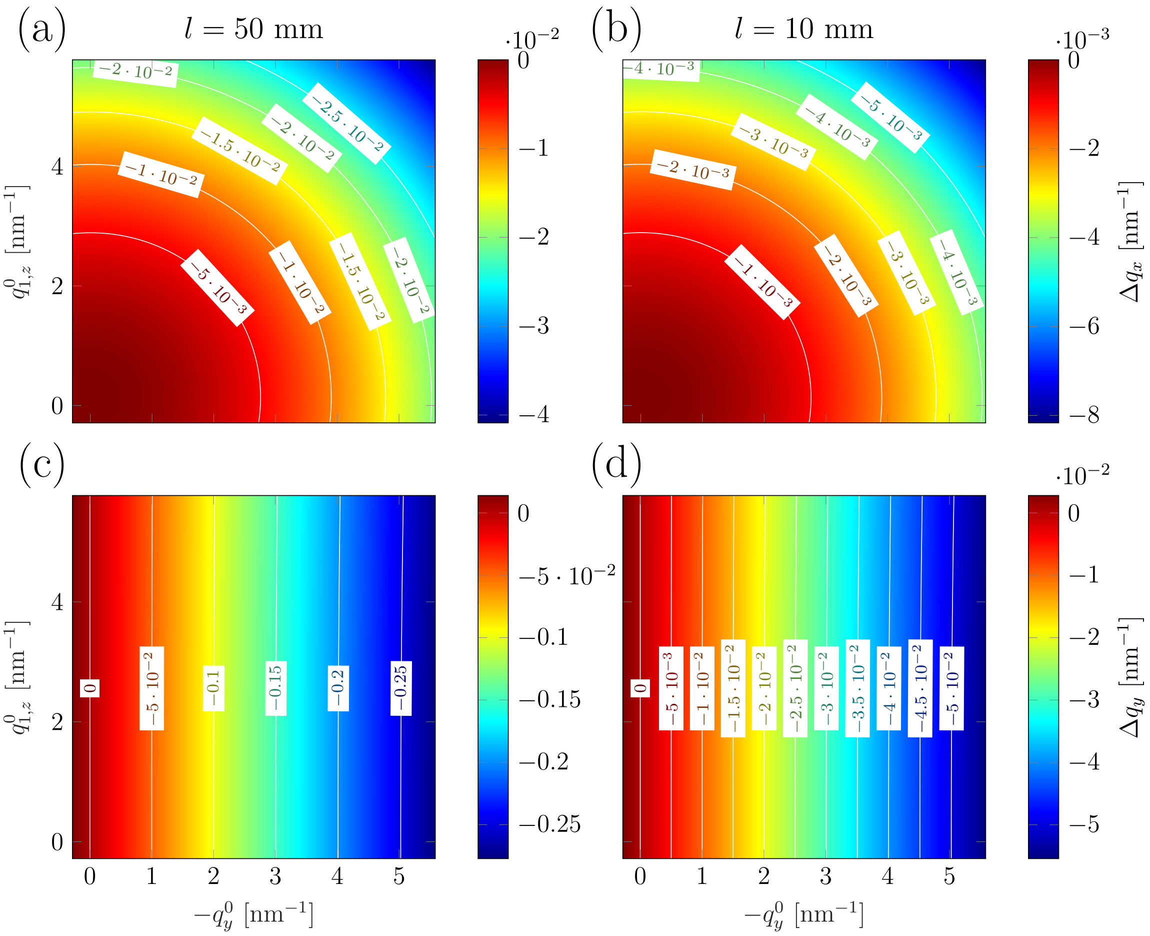

Figure S5: The difference in scattering vector coordinates q_x (a,b) and q_y (c,d) between the edge positions on the flat sample ($X = -l/2$ and $X = l/2$). The left graphs show the data for $l = 50$ mm, whereas the right ones for $l = 10$ mm. The data are calculated for $d = 1$ m and $\alpha_i^0 = 0.1°$.

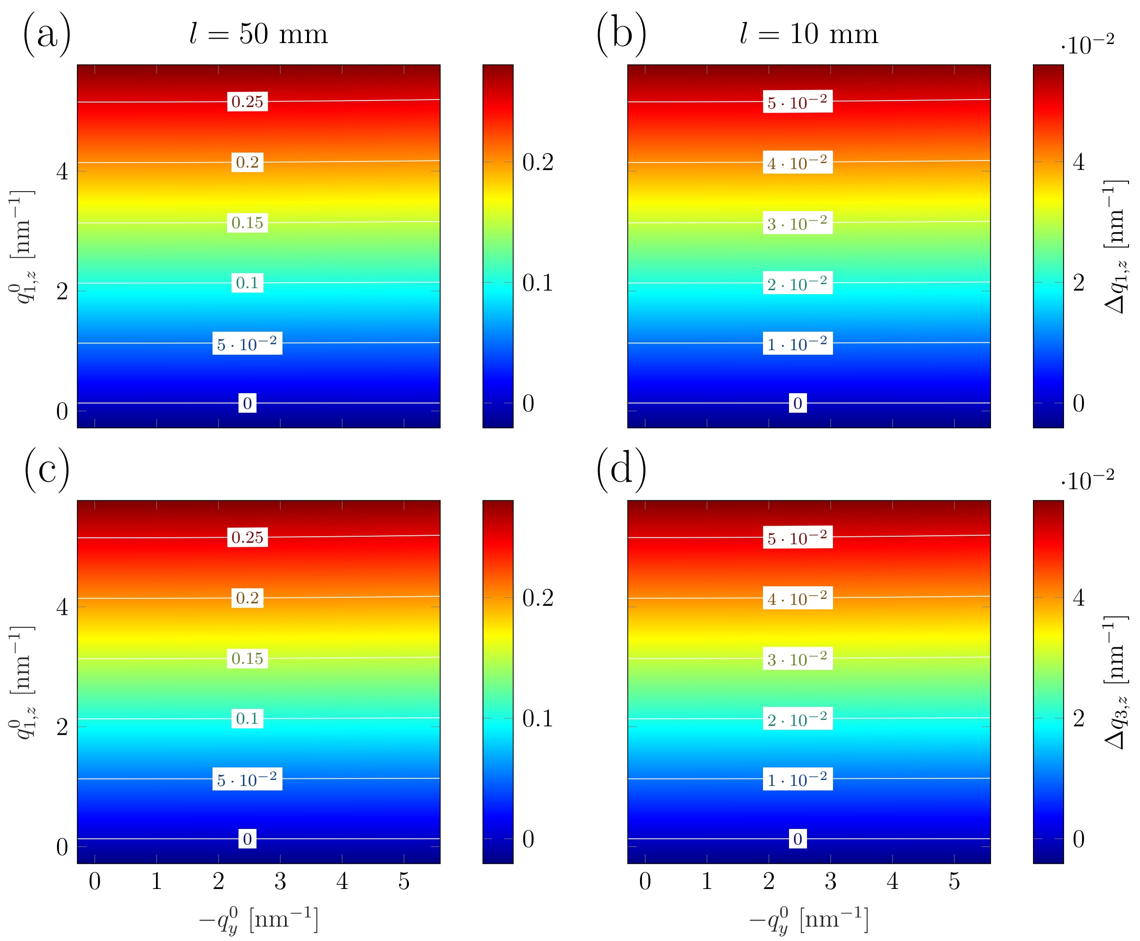

Figure S6: The difference in scattering vector coordinates $q_{1,z}$ (a,b) and $q_{3,z}$ (c,d) between the edge positions on the flat sample ($X = -l/2$ and $X = l/2$). The left graphs show the data for the sample length $l = 50$ mm, whereas the right ones for $l = 10$ mm. The data are calculated for $d = 1$ m and $\alpha^0_i = 0.1°$.

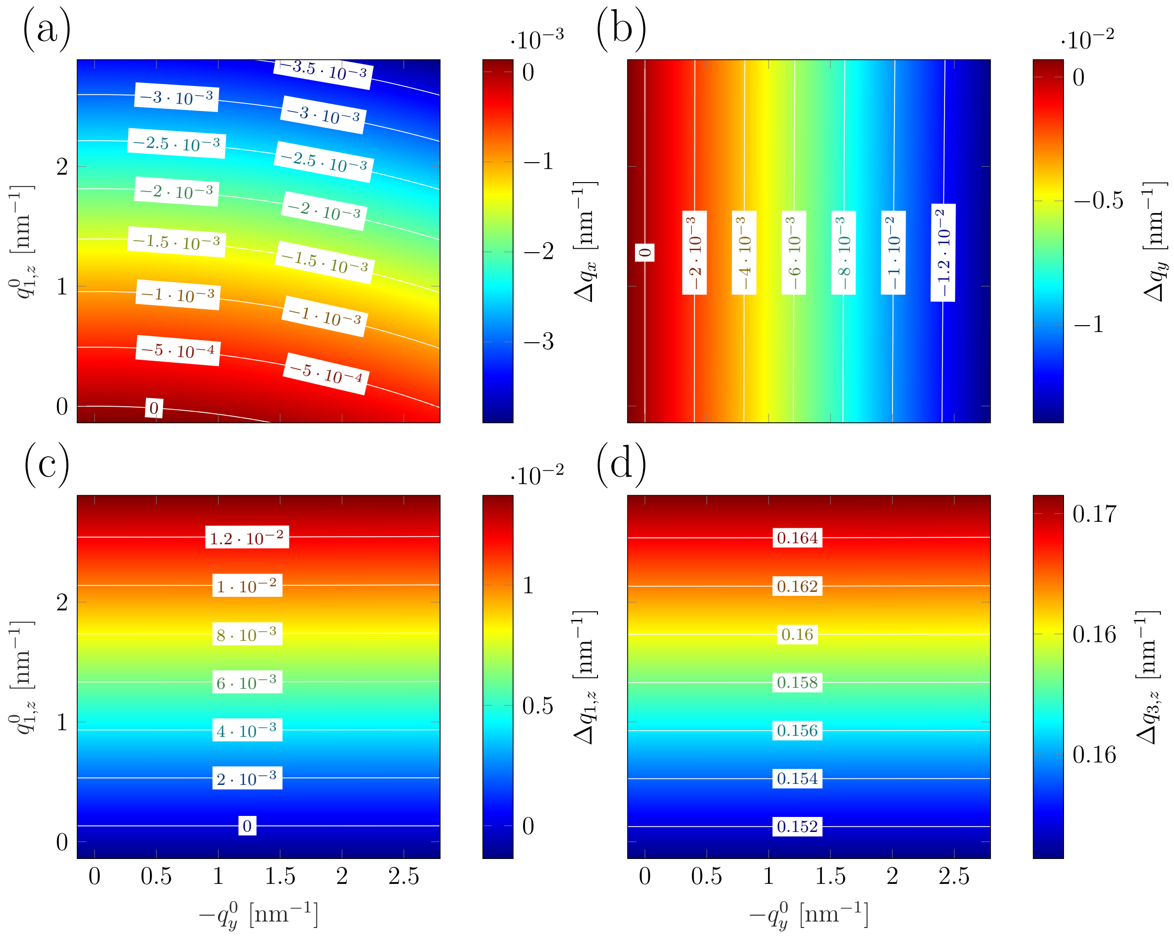

Figure S7: The difference in scattering vector coordinates: (a) q_x, (b) q_y, (c) $q_{1,z}$ and (d) $q_{3,z}$; between the edge positions on the curved sample ($X = -l/2$ and $X = l/2$). The data are obtained for parameters: $l = 10$ mm, $d = 2$ m, $R = -10$ m, and $\alpha_i^0 = 0.1°$.

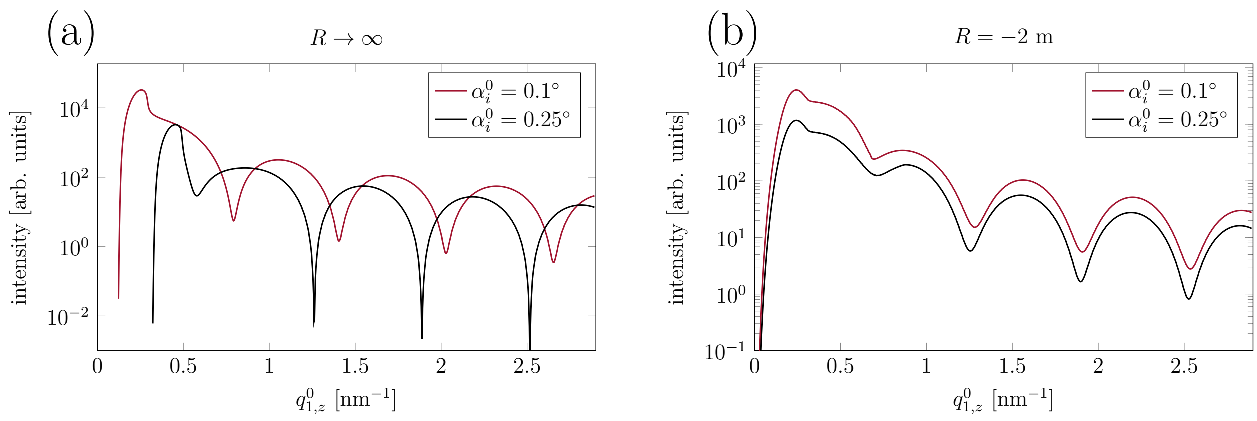

Figure S8: The vertical cuts at $q_y^0 = 0$ through the GISAXS pattern for different nominal incident angles: 0.1° and 0.25°. (a): Reference curves for the flat sample ($R \to \infty$) and (b): curves for the convex sample with $R = -2$ m. One can see that the curvature effect suppresses the dependence on the nominal incidence angle. The calculations were performed for $l = 20$ mm and $d = 2$ m.

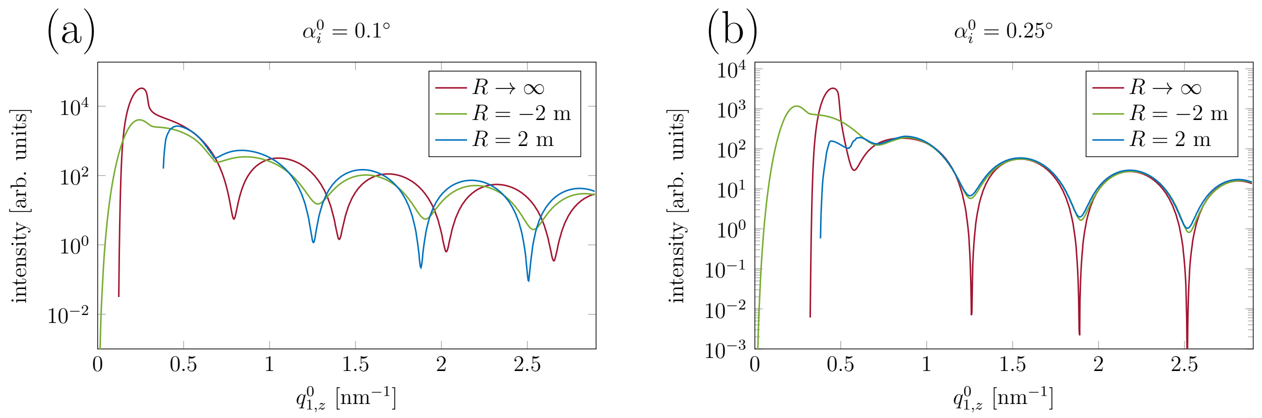

Figure S9: Comparison of the vertical cuts at $q_y^0 = 0$ through the GISAXS pattern showing the impact of curvature effect depending on the nominal incident angle. (a): Curves for $\alpha_i^0 = 0.1°$ and (b): $\alpha_i^0 = 0.25°$. One can see that for higher nominal incident angles the curvature effect is less significant, particularly in higher $q^0_{1,z}$ region. The calculations were performed for $l = 20$ mm and $d = 2$ m.
